# A Randomized, Double-Blind, Placebo-Controlled Trial of Lessertia frutescens in Healthy Adults

**DOI:** 10.1371/journal.pctr.0020016

**Published:** 2007-04-27

**Authors:** Quinton Johnson, James Syce, Haylene Nell, Kevin Rudeen, William R Folk

**Affiliations:** 1 South African Herbal Science and Medicine Institute, University of the Western Cape, Bellville, South Africa; 2 The International Centre for Indigenous Phytotherapy Studies, University of the Western Cape, Bellville, South Africa; 3 Tiervlei Trial Centre, Karl Bremer Hospital, Bellville, South Africa; 4 School of Health Professions, University of Missouri-Columbia, Missouri, United States of America; 5 School of Medicine, University of Missouri-Columbia, Missouri, United States of America

## Abstract

**Objectives:**

Indigenous medicines are widely used throughout Africa, despite a lack of scientific evidence for their safety or efficacy. The aims of this study were: (a) to conduct a pilot study of the safety of a common indigenous South African phytotherapy, *Lessertia frutescens (Sutherlandia)*, in healthy adults; and (b) to contribute to establishing procedures for ethical and scientifically rigorous clinical trials of African indigenous medicines.

**Design:**

A randomized, double-blind, placebo-controlled trial of *Sutherlandia* leaf powder in healthy adults.

**Setting:**

Tiervlei Trial Centre, Karl Bremer Hospital, Bellville, South Africa.

**Participants:**

25 adults who provided informed consent and had no known significant diseases or allergic conditions nor clinically abnormal laboratory blood profiles during screening.

**Intervention:**

12 participants randomized to a treatment arm consumed 400 mg capsules of *Sutherlandia* leaf powder twice daily (800 mg/d). 13 individuals randomized to the control arm consumed a placebo capsule. Each participant received 180 capsules for the trial duration of 3 mo.

**Outcome Measures:**

The primary endpoint was frequency of adverse events; secondary endpoints were changes in physical, vital, blood, and biomarker indices.

**Results:**

There were no significant differences in general adverse events or physical, vital, blood, and biomarker indices between the treatment and placebo groups (*p* > 0.05). However, participants consuming *Sutherlandia* reported improved appetite compared to those in the placebo group (*p* = 0.01). Although the treatment group exhibited a lower respiration rate (*p* < 0.04) and higher platelet count (*p* = 0.03), MCH (*p* = 0.01), MCHC (*p* = 0.02), total protein (*p* = 0.03), and albumin (*p* = 0.03), than the placebo group, these differences remained within the normal physiological range, and were not clinically relevant. The *Sutherlandia* biomarker canavanine was undetectable in participant plasma.

**Conclusion:**

Consumption of 800 mg/d *Sutherlandia* leaf powder capsules for 3 mo was tolerated by healthy adults.

## Editorial Commentary

**Background:** In Africa, traditional herbal medicines are given for many illnesses. In particular, one herbal medicine, *Sutherlandia (Lessertia frutescens)* is commonly given in the belief that this herb will treat some of the symptoms associated with HIV/AIDS, such as nausea and lack of appetite, amongst others. However, there is very little evidence relating to the safety and none to the efficacy of this herb. Generally, when new drugs are developed, the first stage of human testing involves a Phase 1 trial. This type of trial would typically involve small numbers of healthy individuals, who would receive progressively increasing doses of the drug under study, and would be closely monitored for any sign of side effects. Phase 1 trials would typically also collect data from blood samples to find out how the drug is handled in the body and broken down and eliminated. Therefore, the researchers here carried out a preliminary study to assess just the safety of *Sutherlandia*. 25 healthy adults were randomized to receive either tablets containing a fixed dose of *Sutherlandia* leaf powder daily for three months, or matched placebo tablets containing lettuce leaf powder, for the same period of time. The main aim of the trial was to assess safety, so the primary outcomes were adverse events experienced by the participants. The researchers also measured standard outcomes such as blood pressure, heart rate, body weight, urine glucose, protein, and many others, at one-month intervals over the three-month period.

**What the trial shows:** Adverse events experienced by trial participants over the three months of this trial included those that might be expected in a group of otherwise healthy individuals, such as headaches, insomnia, allergies, malaise, palpitations, nosebleeds, and so on. The researchers did not see statistically significant differences between treatment and placebo groups in any of the major categories of these events. Most physical and laboratory measurements also showed no statistically significant differences between the study groups. However, there were statistically significant, but small, differences between groups in respiratory rate and in various basic blood tests. The researchers did not think these differences were clinically important. Overall, this trial suggested that *Sutherlandia* use was not associated with side effects at this dosage and over this time scale.

**Strengths and limitations:** Strengths of this study include the use of randomization to distribute individuals to either the *Sutherlandia* or control groups, and in the use of a placebo control group, which therefore allowed the researchers to compare the frequencies of adverse events in the *Sutherlandia* group with what might be expected among healthy individuals over the course of three months. An important limitation is the small sample size of the trial. This size limits the sensitivity of the trial to detect rare adverse events to the herb under study, and therefore one cannot say conclusively that the herb is safe, based on this data. Additionally, the study looked only at the participants' response to one dosage level of *Sutherlandia*. A strategy using progressively increasing doses would have allowed the researchers to see if there was a maximum tolerated dose to this herb. A further limitation in this study is the lack of data relating to how the herb is broken down in the body; these data are normally an important part of Phase 1 trials and, combined with safety data, are crucial to finding out whether a compound is safe when given at a dosage that allows it to be available to the appropriate tissues.

**Contribution to the evidence:** Data from previous studies in nonhuman primates have shown that *Sutherlandia* is not associated with toxic or other side effects at approximately equivalent or higher doses than that normally taken by people with HIV/AIDS. This study adds safety data relating to *Sutherlandia* consumption in healthy humans, which confirm the primate data. However, it is crucial to collect more data relating to how the probable active ingredients of *Sutherlandia* are absorbed and broken down, and to assess safety at different dosages, before studies are even considered for the next stage, which is to see whether *Sutherlandia* has any efficacy in people with HIV/AIDS.

## INTRODUCTION

The vast majority of people in South Africa use traditional medicines, which the government has recently recognized as an integral part of the public health system. However, there have been no scientific studies of traditional medicines to evaluate their safety and efficacy, nor is there agreement as to the ethical and regulatory norms for the conduct of clinical trials of such phytotherapies.

Infusions and stem and leaf decoctions of the indigenous South African plant Lessertia frutescens (L.) Goldblatt & J. C. Manning (syn. *Sutherlandia frutescens [L.] R. Br.*), commonly called *Sutherlandia,* have been widely used in South Africa as traditional medicines since they were first adopted by the Khoi, San, and Nama peoples. In fact, the traditional Tswana name “Phetola” means “it changes” many illnesses into favorable outcomes. More specifically, it is taken to treat symptoms associated with AIDS [1–3], and to combat cancer [4–6], infections [7], inflammation [8,9], and stress [10].

A study in vervet monkeys *(Chlorocebus aethiops)* found that up to nine times the advertised dose of *Sutherlandia* (81 mg/kg body weight per day for 3 mo) resulted in no significant changes to relevant haematological, biochemical, and physiological parameters [11]. The present investigation aimed to (a) monitor adverse events in healthy human participants; (b) monitor changes in physical, vital, blood, and biomarker indices; (c) contribute to establishing procedures for the ethical and scientific conduct of a human clinical trial of a traditional medicine.

### Objective

Our objective was to conduct a pilot study of the safety of *Sutherlandia* leaf powder capsules in healthy adults.

## METHODS

### Participants

The study took place at Tiervlei Trial Centre, Karl Bremer Hospital, Bellville, South Africa. Forty-one adults were recruited and screened from August to September 2004. Participant inclusion criteria were body weights within 25% of an appropriate body mass index and no significant diseases or clinically significant abnormal laboratory values during screening. Participants had no history of allergic conditions (asthma, urticaria, eczema; autoimmune disorders), systemic lupus erythematosis; dyspepsia, gastric ulcer, or duodenal ulcer; or psychiatric disorders. Their 12-lead ECGs had no significant abnormalities. They were not on regular medical treatment, and did not take any medication 14 d prior to the study. They had a smoking history or less than ten pack-years, no recent history of alcoholism (>2 y) or consumption of alcohol within 48 h of receiving study medication, and did not use any recreational drugs or have a history of drug addiction. Female participants were tested and found not to be pregnant, and all women were requested to use appropriate means of contraception. Participants who did not meet the aforementioned criteria were excluded from the study. All participants were able to communicate effectively with study personnel, were informed of the nature of the study, and provided informed consent.

The study protocol (protocol number M03/11/06) was approved by the Stellenbosch University Institutional Research Board (IRB) on 10 March 2004 with assurances to provide amendments to the board regarding any changes to the protocol, report unexpected serious adverse events or adverse drug reactions suspected to be related to the study drug, notify in the event the study was discontinued, adhere to the principles of informed consent for all participants (providing patient information and consents in English, Afrikaans, and Xhosa languages), and provide progress reports twice each year during the trial. The protocol was also approved by the South African Medicine Control Council (MCC protocol number TICIPS001) on 20 July 2004, with assurances that sufficient amounts of product of assayed quality to conduct the trial was available, and to notify the MCC of any severe adverse events or if the trial was discontinued, provide progress reports during the trial, and to administer the medicine under the direction of an authorized trialist. To ensure medical coverage for potential harm of the individuals from participation in the study trial, the trial was underwritten by a national (South African) insurance company as a requirement by both the Stellenbosch IRB and MCC. Application materials, including the protocol, were provided to the University of Missouri Health Sciences IRB, reviewed and approved on 7 April 2004 (project number 1042077) for conduct of activities in South Africa as approved by the local Stellenbosch IRB and South African MCC reviewing agencies.

### Interventions

**Figure 1:**
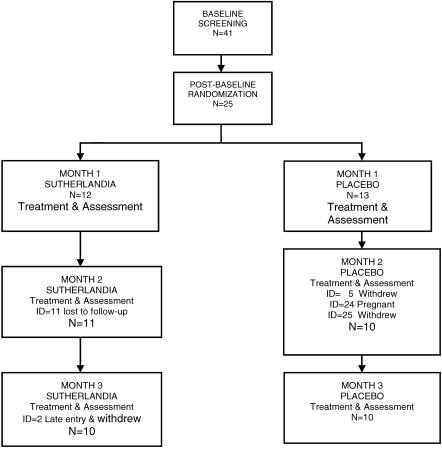
Trial Profile CONSORT flowchart

Of the 41 participants, 25 who met the trial criteria were enrolled in the study and randomized to two groups (Figure 1): 12 randomized to a treatment arm consumed capsules containing 400 mg of *Sutherlandia* leaf powder (400 mg of plant material per capsule; 600 μg of canavanine per capsule) twice daily (800 mg/day) and 13 randomized to the control arm consumed a placebo capsule of lettuce leaf powder twice per day. The treatment and placebo materials were placed in rapidly releasing capsules, and these were assessed pharmaceutically for content uniformity, stability, and release characteristics as well as microbial, heavy metal, and pesticide contamination, before the products were packaged and used in the 3-mo clinical trial. Furthermore, the clinical trial conformed to MCC and NCCAM guidance on the quality of biologically active ingredients and placebo materials used in complementary, alternative, and traditional medicine products.

### Outcomes

The primary endpoints of this study were incidence (number) and type of adverse events recorded during the treatment period.

The secondary endpoints were: (a) changes in weight, blood pressure, heart rate, respiratory rate, body temperature from baseline to end of treatment; and (b) changes in haematological and biochemical parameters from baseline to end of treatment.

### Sample Size

In the absence of previous data, a convenience sample size of about 12 participants per treatment group was chosen for this first study of a South African traditional medicine in healthy humans. This relatively small sample size is a limitation of the study. It does, however, give sufficient power to detect an effect size of 1.20 with 80% power based on doing a two-sample t-test at a significance level of 0.05 with a two-sided alternative. Effect size is defined as the difference in group means divided by the standard deviation of the response variable under consideration. This applies to numeric variables. For categorical variables, the numbers are too small to do meaningful tests.

### Randomization: Sequence Generation

To achieve randomization, a list of random numbers allocating to the two treatments (A, active treatment; B, placebo) a maximum of 13 participants per group was generated using a GraphPad Software (http://www.graphpad.com) calculator option. For each number, a 3-mo supply of the relevant treatment (active or placebo) was labeled with that particular number. The randomized number generation and labeling of the treatments were performed by one person at the School of Pharmacy, University of the Western Cape, and the labeled materials (for 3-mo dosing of each of 25 participants) shipped to the clinical trial site.

### Randomization: Allocation Concealment

The person generating the randomized list also did the labeling of the treatments and kept the randomized list.

### Randomization: Implementation

At the trial site, participants were sequentially allocated to the treatments in the order in which they were recruited, i.e., the first person who qualified for inclusion was given treatment number 1, the second one treatment number 2, and so on. When allocated, the participant ID number was added to the label details on the capsule containers. The clinician on site made this allocation.

### Blinding

The placebo and treatment capsules containing plant materials were packed in identical nontransparent containers. Neither the participants nor the clinicians knew which treatment they received or dispensed. The data collected were retrieved from the case report form by another researcher, at the South African Herbal Science and Medicine Institute, the University of the Western Cape, who loaded it into a database. Once accuracy of the data was confirmed (i.e., clean file status), the database was forwarded to the statisticians who, only at this time, were supplied with the randomized treatment list. The statisticians then identified the participants allocated to each treatment.

### Laboratory Measurements and Blood Sampling

Participants were provided with diaries in which to self-report all adverse events for the duration of the trial. Participants were screened (at the baseline visit, participants were screened for physical, vital, haematological, biochemical, and endocrine indices), randomized (at postbaseline, participants were provided with capsules containing either *Sutherlandia* or placebo material), treated, and assessed (at months 1–3) for the same parameters that were determined at the baseline visit. Participants were subjected to a physical examination (weight, blood pressure, heart rate, respiratory rate, body temperature, and ECG measurement), and they provided blood samples for haematology, endocrine, and biochemistry analysis by standard laboratory methods. The plasma samples of trial participants were analyzed for canavanine by liquid chromatographic separation and mass spectrophotometric determination, with a Waters API Q-TOF Ultima LCMSMS system (http://www.waters.com). Other biochemical variables were measured with Abbott AxSYM (http://www.abbottdiagnostics.com) and Beckman Coulter CX9 ALX (http://www.beckmancoulter.com) systems, respectively. All haematology indices were measured using a Beckman Coulter Gen-S system, while CD3, CD4, and CD8 counts were determined by flow cytometry, with a Beckman Coulter EPICS system.

### Statistical Methods

Analysis for adverse events was done descriptively. With the small number of participants in each group the power to detect all but very large differences is relatively small. Doing Chi-square tests and finding that results are insignificant would not be very informative. Consequently, the number of participants who experienced adverse events of different types is given. Some participants reported the same adverse event (e.g., “increased appetite”) on more than one occasion. For this reason we counted the number of participants who experienced an adverse event at least once for the summary rather than giving a count of the number of times the event was reported. Counts, proportions, and 95% confidence intervals for the proportions are given.

For the statistical analysis of all the haematology and biochemistry parameters, a repeated measures ANOVA model was used, with the treatment group as one factor and PREPOST, an indicator for the post-treatment measurement, as a factor with repeated measures. The interaction term (treatment by PREPOST) measures the difference between the comparison of *Sutherlandia* (treatment) versus placebo groups at PRE (baseline) and the comparison of *Sutherlandia* versus placebo groups at POST (months 1–3: treatment). The Kenward-Roger denominator degrees of freedom method was used for the fixed effects testing, whilst Proc Mixed in SAS Version 9.1 (http://www.sas.com) was used for the modelling. Least squares means were used to estimate treatment effects at the combined visits 3–5 based on the mean response models. Comparisons between pretreatment and post-treatment were obtained for all indices in the study. Statistical significance for these outcomes was set at the 5% level. Again, in the spirit of being conservative, adjustments for multiple tests (such as the Bonferroni adjustment) were not made. This most likely resulted in some false positives.

## RESULTS

### Recruitment and Participant Flow

A total of 41 participants were recruited and screened (visit 1: baseline physical, vital, and blood indices) between August and September 2004. Of these, 25 consented, met the trial criteria, and were subjected to a blood draw for haematology, endocrine and biochemistry analysis, to establish baseline (pretreatment) values. Thereafter, they were randomized (postbaseline) to receive treatment capsules (*n* = 12) or placebo control capsules (*n* = 13) that contained a small amount of dried lettuce leaves twice per day, treated, and assessed (at months 1–3). Participants were given a diary to record the times they took their trial medication, adverse effects they may have experienced, and other medications they may have taken over the course of the trial. During the three-month study period, one adult was lost to follow-up and another withdrew from the treatment group. In the placebo group, two adults withdrew and another became pregnant.

### Baseline Data

Baseline data for vital, physical, haematological, biochemical, and endocrine indices were similar for the 25 eligible participants (Table 1).

**Table 1:**
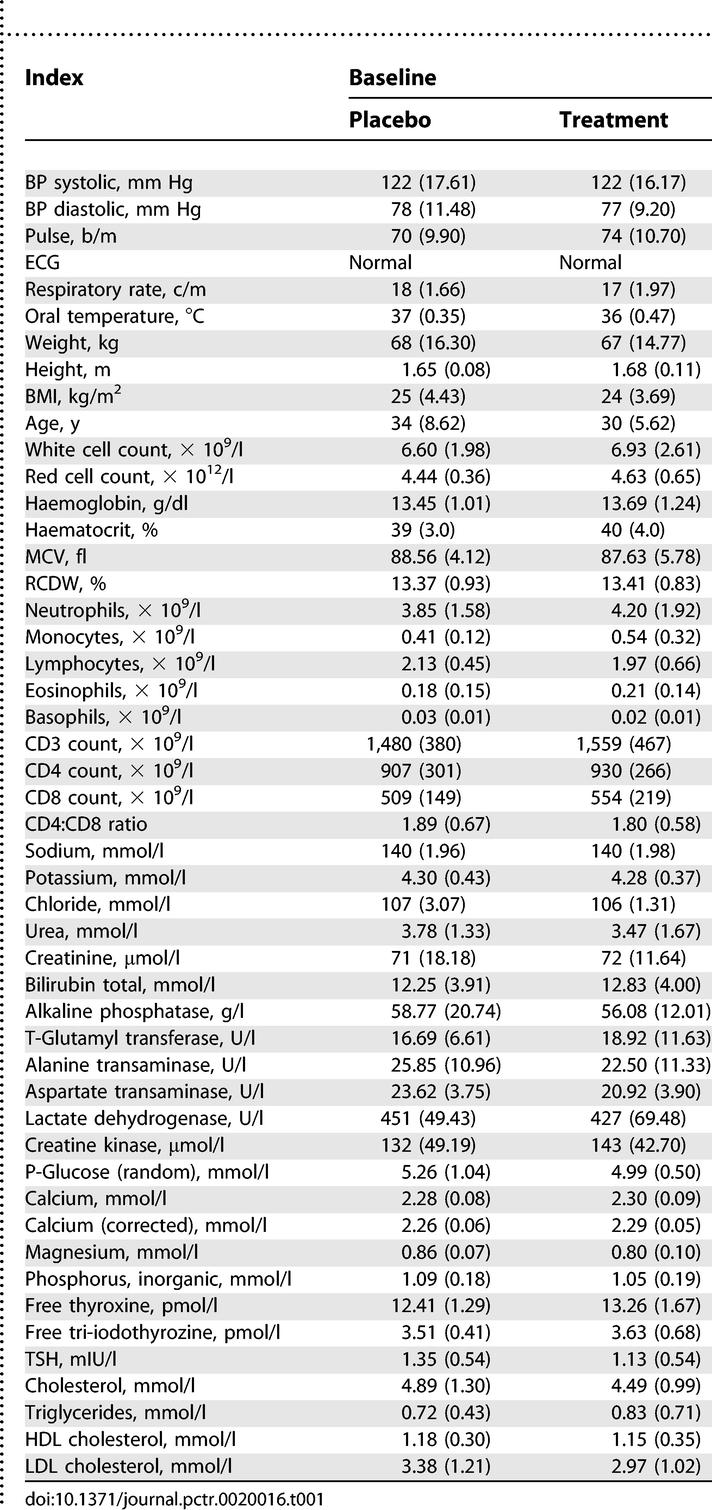
Baseline Data (Mean ± Standard Deviation) for Trial Participants

### Outcomes and Estimation

In summarising the adverse events reported by participants at any of the follow-up visits, we note that one individual in the placebo group was only in the study for 5 d, with no follow-up data. That individual is not included in the summary given so there are 12 participants in each group. While the number of days in the study did vary from participant to participant, the cumulative number of exposure days for the two groups were similar, with 957 and 972 total days for the treatment and placebo groups, respectively. A count was made of the number of participants who reported a particular type of adverse event at least once. The results are summarized in Table 2, where, in addition to the counts, the percentage of participants (out of 12) who experienced the event at least once is given along with an exact 95% confidence interval estimate of the proportion. As shown in the table, the types of events include cardiovascular (e.g., palpitations, nosebleeds), central nervous system (CNS; e.g., headaches, nervousness, insomnia, dizziness), gastrointestinal tract (GIT), infection, allergy, appetite, malaise, or general adverse events. The last line of Table 2 gives the number of participants who reported any adverse event at any time. We would point out that with the small number of participants, it is unlikely that rare adverse events would be seen in this study.

**Table 2:**
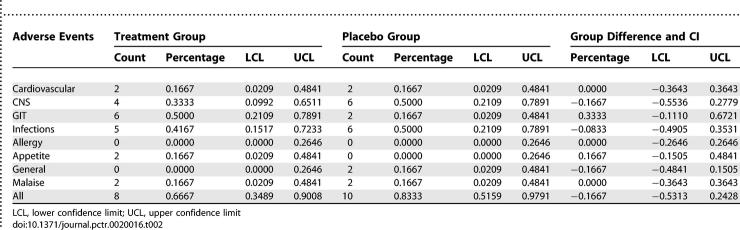
Counts, Percentages, Group Differences and 95% Confidence Interval Estimates of Proportion of Subjects Reporting at Least One Adverse Event of the Type Described

Table 3 provides a list of the vital, physical, haematological, biochemical, and endocrine data that were not significantly different between the treatment and placebo groups (*p* > 0.05).

**Table 3:**
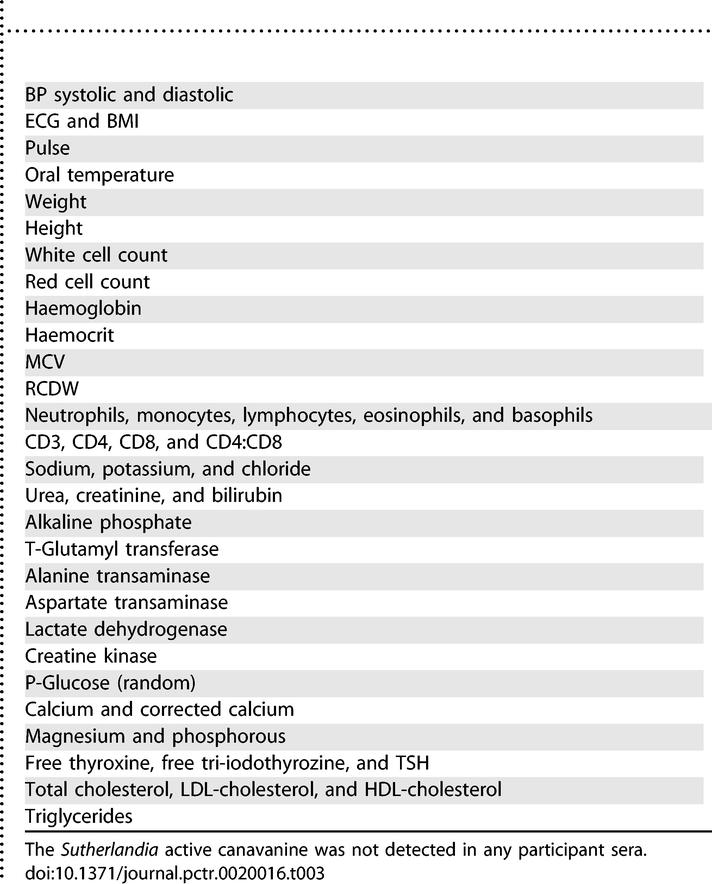
Vital, Physical, Haematological, Biochemical, and Endocrine Endpoints that Were Not Significantly Different between the Treatment and Placebo Groups (*p* > 0.05), and within the Normal Physiological Range for Humans

Most of the vital and physical, haematological, biochemical, and endocrine endpoints that were measured were within the normal physiological range and not significantly different for the *Sutherlandia* and placebo groups. These include: diastolic and systolic blood pressure (BP), electrocardiogram (ECG), heart rate, body temperature (oral), and weight and height; white cell and red cell count**s**, haemoglobin, haematocrit, mean corpuscular volume (MCV), and red cell diameter and width (RCDW); neutrophil, monocyte, lymphocyte, eosinophil, and basophil counts; CD3, CD4, CD8 counts, and CD4:CD8 ratio; sodium, potassium, and chloride; urea, creatinine, and bilirubin; alkaline phosphatase, T-glutamyl transferase, alanine transaminase, aspartate transaminase, lactate dehydrogenase, creatine kinase, plasma glucose (random), calcium and corrected calcium, magnesium, and phosphorous; free thyroxine, free tri-iodothyroxine, and TSH; and cholesterol, LDL-cholesterol, HDL-chlolesterol, and triglycerides. No canavanine was detected in any of the samples.

Table 4 contains the data for the six variables that were statistically different between the treatment and placebo groups (*p* < 0.05). The *Sutherlandia* group had a lower respiration rate (*p* < 0.04), but higher platelet count (*p* = 0.03) than the placebo group. In relation to baseline values, mean corpuscular haemoglobin (MCH; *p* = 0.01) and mean corpuscular haemoglobin concentration (MCHC; *p* = 0.02) levels were lower for the *Sutherlandia* compared to placebo group.

**Table 4:**
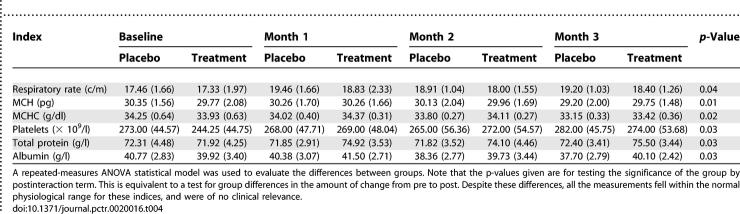
Vital, Physical, Haematological, and Biochemical Endpoints (Mean ± Standard Deviation) that Were Significantly Different between the Treatment and Placebo Groups (*p* < 0.05), and within the Normal Physiological Range for Humans

In addition, the *Sutherlandia* group had higher total protein (*p* = 0.03) and albumin levels (*p* = 0.03) than the placebo group.

Note that the *p*-values given are for testing the significance of the group by post interaction term. This is equivalent to a test for group differences in the amount of change from pre-treatment to post-treatment. Despite these differences, all the measurements fell within the normal physiological range for these indices, and were of no clinical relevance.

## DISCUSSION

### Interpretation

This pilot study is the first to provide scientific information on the safety of the South African traditional medicine *L. frutescens (Sutherlandia)* in healthy humans, and contribute to establishing rigorous and ethical procedures for conducting clinical trials on indigenous phytotherapies. *Sutherlandia* is used for a variety of conditions, including those associated with AIDS. Analysis for adverse events was done descriptively and included: cardiovascular (palpitations, nosebleeds), CNS (headaches, nervousness, insomnia, dizziness), GIT, infection, allergy, appetite, malaise, or general adverse events. With the small number of participants, which is a limitation to the study, it is unlikely that rare adverse events would be seen.

There were no significant differences in cardiovascular, CNS, GIT, infection, allergy, malaise, or general adverse events between the treatment and the placebo groups. Whilst participants in the treatment group experienced more events related to appetite, the constraints of the investigation related to limited sample size precludes firm conclusions from being drawn about these preliminary data or any speculation related to mechanisms of action. It is nonetheless interesting to have observed this outcome, since *Sutherlandia* is purported to prevent wasting, and is in contrast to the report that cachectic patients consuming *Sutherlandia* exhibited side effects such as diarrhea, mild diuresis, dry mouth, and dizziness [12].

There were no significant differences between the treatment group and placebo group for most of the vital, physical, haematological, biochemical, or endocrine parameters. In adjusting for baseline values, the treatment group had higher MCH, MCHC, platelet, total protein and albumin count, and lower respiration rate compared to the placebo group, but these differences were not clinically significant. Whilst the latter outcome is noted, we do not have any dose-escalation or pharmacokinetic data from this pilot study, in relation to *Sutherlandia* and its effect on vital, physical, haematological, biochemical, and endocrine indices, which is a limitation of this investigation.

### Generalizability

*Sutherlandia* in the amount studied (800 mg/day) is widely advertised as being safe and is undoubtedly consumed by many, and it was tolerated by the limited number of healthy adults in this pilot study. Nevertheless, additional studies are warranted to assess the safety and efficacy of this and other phytotherapies.

This study has established a precedent for the ethical and scientifically rigorous evaluation of indigenous medicines used by the public, and it is hoped that additional studies will quickly follow.

### Overall Evidence

Until now, no human studies have been conducted to assess the safety or efficacy of *L. frutescens (Sutherlandia)* with its claimed benefits ascribed to constituents including pinitol, γ-amino butyric acid (GABA), and L-canavanine [13,14]. It is not known if these constituents work in isolation or in unison. GABA is an inhibitory neurotransmitter present in appreciable quantities [14], and it may potentially have beneficial effects on stress, anxiety, and depression, which adversely affect the course of HIV disease [15]. Canavanine is a potent L-arginine antagonist, with antiviral, antifungal, antibacterial, and anticancer value [16–19]. Concerns have been raised over the possible induction by canavanine of autoimmune diseases (such as systemic lupus erythematosis), which is thought only to occur at very high doses in predisposed individuals or in the presence of low arginine levels [20]. However, canavanine was not detected in the plasma of the trial participants, perhaps because of the dosage and its metabolism, and possible biotransformation into another molecular entity through the P450 system, which is affected by L. frutescens [21].

A recent Cochrane review of nine randomised placebo-controlled trials involving 499 individuals with HIV infection and AIDS, wherein eight different Chinese herbal medicines were tested, was recently completed [22]. Evidence for the effect of the eight herbal medicines identified in the review for treatment of HIV infection and AIDS was not compelling. Moreover, the review concluded that a need exists for larger and more rigorously designed trials. To our knowledge, this is the first report of a double-blind, randomized, placebo-controlled study assessing the safety of the African indigenous medicine *Sutherlandia*. This investigation provides a vital step for better understanding the clinical value of traditional phytotherapies in healthy humans. Given that this African indigenous medicine is so extensively used [23], and is taken by many for the treatment of a variety of conditions including those associated with HIV and AIDS, additional safety and efficacy trials are warranted in the interest of public health.

## SUPPORTING INFORMATION

CONSORT Checklist (43 KB DOC)

Trial Protocol (357 KB DOC)

## Abbreviations

CNS: central nervous system
GIT: gastrointestinal tract
MCH: mean corpuscular haemoglobin
MCHC: mean corpuscular haemoglobin concentration
MCV: mean corpuscular volume
RCDW: red cell diameter and width
