# Supplementary material for: A Randomized, Double-Blind, Placebo-Controlled Trial of Lessertia frutescens in Healthy Adults
Source: PLoS Clin Trials. 2007 Apr 27;2(4):e16. doi: 10.1371/journal.pctr.0020016 (PMC1863514; doi:10.1371/journal.pctr.0020016)
Supplement: Trial Protocol — (357 KB DOC) [file pctr.0020016.sd002.doc]

# Protocol Cover Page

**Protocol Title:** A randomized double blind placebo controlled study to investigate the safety of *Sutherlandia frutescens (subspecies microphylla)* in healthy adult volunteers.

**Protocol Number:** TICIPS001

**Protocol Date:**  10 October 2003

**Study Phase:** I

**Project Leader:** J A Syce

**Protocol Author(s):** J A Syce PhD

H Nell MBChB

G Fatti MBChB

#### **SPONSOR**: ***TICIPS***

The International Centre for Indigenous Phyotherapy Studies

South African Herbal Science and Medicine Institute, University of the Western Cape (UWC), Private Bag X17, Bellville, South Africa.

#### **INVESTIGATOR:**

Dr H Nell,

Tijger Trial Centre (TTC),

1^st^ Floor, Ward 1A,

Mike Pienaar Boulevard, Karl Bremer Hospital,

Bellville 7531,

South Africa.

# Confidentiality Agreement

This document is a confidential communication of TICIPS. Acceptance of this document constitutes the agreement by the recipient that no unpublished information contained within will be published or disclosed without prior written approval, except that this document may be disclosed to the appropriate Ethics Committee and Regulatory Authority under the condition that they are requested to keep it confidential.

# TABLE OF CONTENTS PAGE

LIST OF ABBREVIATIONS AND DEFINITIONS 4

STUDY CONTACT LIST 6

PROTOCOL SYNOPSIS 7

1 INTRODUCTION 9

- 1. Background 9
  2. Study Rationale 10

1. STUDY OBJECTIVES 10
2. STUDY PLAN AND PROCEDURES 10
   1. Study design 10
      1. Scheduled clinic visits 11
      2. Visits and assessments 11
   2. Study population 11
      1. Inclusion criteria 11
      2. Exclusion criteria 12
      3. Justification for inclusion and exclusion criteria 12
      4. Criteria for discontinuation 12
   3. Investigational Products and Treatments 12
      1. Treatment Schedule 12
      2. Randomization 13
      3. Identity of Study Products 13
      4. Storage and Accountability 13
      5. Allowed medication 13
      6. Compliance 13
3. STUDY MEASUREMENTS AND ENDPOINTS 14
   1. Primary Safety Endpoints 14
   2. Secondary Safety Endpoint 14
   3. Measurements at each visit 14
   4. Specific detail on measurements 15
      1. Adverse Events 15
      2. 12-Lead ECG 16
      3. Laboratory Investigations 16
         1. Haematology 16
         2. Clinical Chemistry 16
         3. Blood levels of active constituents 16
   5. Measurements recorded daily in diary 17
4. STATISTICAL METHODS 17
   1. Determination of sample size 17
   2. Statistical analysis
   3. Changes to the protocol 17
5. ETHICS 18
   1. Ethics review 18
   2. Ethical conduct of the study 18
   3. Subject information and consent 18
   4. Subject data protection 18
   5. Insurance and Indemnity 18
6. DATA QUALITY ASSURANCE 18
7. STUDY TIMETABLE AND TERMINATION 19
8. REFERENCES 19
9. APPENDICES
   1. Declaration of Helsinki 21
   2. Sample written informed consent 25
   3. Diary for treatment period 39
   4. Ideal Body Mass Tables 43
      1. Sutherlandia Literature 44

# LIST OF ABBREVIATIONS AND DEFINITIONS

The following abbreviations and specialist terms are used in this study protocol:

### Abbreviation Explanation

AE Adverse Event

ALP Alkaline phosphatase

ALT Alanine aminotransferase

AST Aspartate aminotransferase

b.i.d *bis in die* = twice daily

CD4+ count of CD4 expressing helper T lymphocytes

CD8+ count of CD8 expressing cytotoxic T lymphocytes

CK creatine phosphokinase

CRF Case Report Form

ECG Electrocardiogram

GABA Gamma aminobutyric acid

GCP Good Clinical Practice

GGT gamma glutamyl transpeptidase

Hb Haemoglobin

Hct Haematocrit

HDL-C High Density Lipoprotein –cholesterol

HPLC High performance liquid chromatography

ICH International Conference of Harmonisation

IEC Independent Ethics Committee

IRB Independent Review Board

LCMS Liquid chromatography linked mass spectrometry

LDH Lactate dehydrogenase

LDL-C Low Density Lipoprotein -cholesterol

MS Mass spectrometry

MCH Mean corpuscular haemoglobin

MCHC Mean corpuscular haemoglobin concentration

MCV Mean corpuscular volume

ml milliliter

MRC Medical Research Council

OHRP Office of Human Research Protections (of Federal Department of Health & Human Services, USA)

RDW Red cell distribution width

RBC Red Blood Cells

SAE Serious Adverse Event

SG Specific gravity

SEM Standard error of the mean

SLE Systemic lupus erythematosus

T3 Triiodothyronine

T4 Thyroxine

TICIPS The International Centre for Indigenous Phytotherapy Studies

TSH Thyroid-stimulating hormone

TTC Tijger Trial Centre

UWC University of the Western Cape

### WBC White blood cells

### Definitions

*Herbal Medicines*: Mainly whole, fragmented or cut plants, part of plants in an unprocessed state, usually in dried form, but sometimes fresh. Herbal drugs are precisely defined by the botanical scientific name according to the binomical system (genus, species, variety).

*African traditional medicine:* The total body of knowledge and techniques for the preparation and use of substances, measures and practices that are based on the socio-cultural and religious bedrock of African communities, are founded on personal experience and observations handed down from generation to generation, either verbally or in writing, and are used for the diagnosis, prevention or elimination of imbalances in physical, mental or social well-being.

## STUDY CONTACT LIST

**For questions regarding the conduct of this study, please contact:**

**Project Leader:** James A Syce, M Pharm, PhD

E-mail: [**jsyce@uwc.ac.za**](mailto:jsyce@uwc.ac.za)

Telephone: +27 21 959 2192

Mobile: 082 202 3315

Telefax: +27 21 959 1324

**Clinical Trial Manager:** Haylene Nell, MBChB, BSc (Honns-Pharmacology)

**(Clinical investigator)** E-mail: [**haylenenell@mweb.co.za**](mailto:haylenenell@mweb.co.za) or ttctrials@mweb.co.za Telephone: +27 21 918 1399/ 1272

Mobile: 083 448 1456

Telefax: +27 21 918 1468

**For reporting a serious adverse event, please contact:**

**Clinical Trial Manager:** Haylene Nell, MBChB, BSc (Honns-Pharmacology)

E-mail: [**haylenenell@mweb.co.za**](mailto:haylenenell@mweb.co.za) or ttctrials@mweb.co.za Telephone: +27 21 918 1399/ 1272

Mobile: 083 448 1456

Telefax: +27 21 918 1468

**For questions regarding data quality assurance, please contact:**

# Independent Study Monitor: Ms Laurie Ben-yair, CRA

On Q Consulting, CC.

E-mail: [**laurieb@onqsa.co.za**](mailto:laurieb@onqsa.co.za)

#### Telephone: +27 21 556 1524

Mobile: 083 457 6050

Telephone: +27 21 556 1524

### PROTOCOL SYNOPSIS

| **TITLE** | A randomized double blind placebo controlled study to investigate the safety of *Sutherlandia frutescens* (subspecies *microphylla)* in healthy adult volunteers. | | |
| --- | --- | --- | --- |
| **Investigational site:** | TTC, First Floor Ward 1A, Karl Bremer Hospital, Bellville, Cape Town, South Africa (& UWC, Health Centre, Bellville, Cape Town, South Africa) | | |
| **Investigators:** | Prof. James Syce, Dr. Haylene Nell, Prof. Pierre. Mugabo, and Prof. Quinton Johnson | | |
| **Sponsor:** | The International Centre for Indigenous Phytotherapy Studies (TICIPS) | | |
| **Representatives:** | Prof. Quinton Johnson | | |
| **Study number:** | TICIPS001 | | |
| **Final Protocol:** | Oct 2003 / **19 Feb 2004** | **Clean File:** | August 2004/  **November 2004** |
| **Ethics Approval:** | Nov 2003 / 1**0 Mar 2004**  **MCC approval: 20 Jul 2004** | **Statistical analysis:** | August 2004 /  **November 2004** |
| **Clinical Phase:** | Mar to Jul 2004/  **Aug – Nov 2004** | **Study Report:** | September 2004/ **November 2004** |
| **OBJECTIVES:** | To investigate the safety of *Sutherlandia frutescens* (subsp. *Microphylla*) capsules in healthy adult volunteers by assessing, liver, kidney, muscle, metabolic, intestinal, cardiac and bone marrow function, as well as physiological and physical status. | | |
| **STUDY DESIGN:** | A randomized double blind placebo controlled study over a 3-month period. Volunteers will be screened at Visit 1 and eligible subjects will, at visit 2, be randomized to either *Sutherlandia frutescens* (subsp. *Microphylla*) 1 x 400 mg capsules b.i.d or a placebo capsule of similar appearance. Visits 3, 4 and 5 (final) will be scheduled at monthly intervals. Safety tests will be repeated at each of the study visits (except visit 2). Patients will enter adverse events and daily use of study medication in a subject diary. This will serve as a measure of compliance. | | |
| **SUBJECTS:** | Inclusion criteria Healthy males and females between 18 and 45 years of age will:   - be informed of the nature of the study and will give written informed consent, - have body weights within 25% of the appropriate weight range, - have no significant diseases or clinically significant abnormal laboratory values during screening, - have 12 lead ECG without significant abnormalities, - be on no regular medical treatment, - be able to communicate effectively with study personnel  Exclusion criteria  - Any disease or condition which might compromise the haematopoietic, renal, endocrine, pulmonary, central nervous, cardiovascular, immunological, dermatological, gastrointestinal or any other body system. - History of allergic conditions – asthma, urticaria, eczema. - History of autoimmune disorders – lupus erythematosis (SLE) - History or presence of dyspepsia, gastric ulcer or duodenal ulcer. - History of psychiatric disorders. - Intake of any medication within 14 days before start of the study - Recent history of alcoholism (<2 years) or consumption of alcohol within 48 hours of receiving study medication. - Smokers, who smoke more than 10 cigarettes per day and cannot refrain from smoking during the study period. - Presence of clinically significant abnormal laboratory results during screening. - Pregnancy or not using appropriate means on contraception - Use of any recreational drugs or a history of drug addiction. | | |
| **PRODUCT TO BE EVALUATED** | **Test:** *Sutherlandia frutescens* (subsp.*microphylla)* leaf powder in 2 X 400mg capsules/day.  **Reference:** Placebo 2 X 400mg capsules /day | | |
| **DURATION OF STUDY** | 3 months | | |
| **ENDPOINTS:** | **SAFETY**   1. Adverse events 2. Physical examination, including weight 3. Vital signs: blood pressure, heart rate, respiratory rate, body temperature. 4. ECG 5. Urine dipstix: leucocytes, nitrate, urobilinogen, protein, pH, blood, specific gravity, ketones, bilirubin and glucose 6. Laboratory tests   Haematology: RBC, MCHC, MCH, MCV, RDW, Hct, Hb, WBC Differential count –lymphocytes monocytes, neutrophils, eosinophils, basophils; CD4+, CD+.  Biochemistry: Serum - Bilirubin (total and conjugated) AST, ALT, ALP, GGT, LDH, CK, Protein, albumin, globulin, cholesterol (total, LDL-C, HDL-C), glucose, urea, creatinine, sodium, potassium, chloride, calcium, magnesium.  7. Pregnancy   1. Active constituent levels: Canavanine, GABA, Pinitol. | | |
| **SAMPLE** **SIZE:** | 24 subjects | | |
| **STATISTICAL ANALYSIS**: | Summary statistics will describe the safety variables. Significance testing will be done using analysis of variance for continuous and chi-square test for categorical variables. (α = 0,05) | | |

# 1. INTRODUCTION

- 1. **Background**

*Sutherlandia frutescens (subspecies microphylla)* (L.) R.Br. is thought to be among the most profound and useful of the medicinal plants in Southern Africa (van Wyk & Gericke, 2000). Tinctures, infusions and decoctions of the leaves and young stems have been widely used in the Cape Region, since the value of this herbal medicine was first discovered by the Khoi, San and Nama people (van Wyk, van Oudtshoorn & Gericke, 2000). The traditional Tswana name, Phetola, means, “it changes”. When literally translated, it means that the plant changes the course of many illnesses into favorable outcomes.

Furthermore, the North Sotho name, Lerumo-lamadi, means “the spear for the blood” which refers to the value of *S. frutescens* as a powerful blood-purifier that acts as an all-purpose tonic ([**www.astral-natural.com/Africana.htm**](http://www.astral-natural.com/Africana.htm)**,** appended). Over centuries, this medicinal plant has been indigenously used to treat a variety of conditions including, cancer, viral, bacterial, fungal and parasitic infections, menopausal conditions, varicose veins and hemorrhoids, inflammation, wasting and stress (van Wyk & Gericke, 2000). The medicinal value of the plant has been ascribed to a number of the significant constituents of this phytotherapy, including pinitol, γ-amino butyric acid (GABA), asparagine, sugaryl (SU1) and also L-canavanine.

Pinitol, which is a known anti-diabetic agent (Davis *et al.*, 2000, Larner *et al*, 1998), has been isolated from *S. frutescens* leaves. Researchers suggest that pinitol may have clinical application in treating the wasting in cancer and AIDS patients (van Wyk & Gericke, 2000). Pinitol is also claimed to have anti-inflammatory effects (Singh et al 2001). Some *S. frutescens* subspecies have high levels of concentrations of GABA, an inhibitory neurotransmitter that is assumed to account for their use for anxiety, stress and depression. In *S. frutescens*, asparagine could function as a diuretic by stimulating the kidneys and liver (Rosenthal, 1992). Sugaryl (SU1), a novel triterpenoid glucoside, has only recently been identified, and was used as a marker to select elite *Sutherlandia* chemotypes for the propagation of the plant raw materials ([**www.sutherlandia.org/chemistry.html**](http://www.sutherlandia.org/chemistry.html), appended).

Canavanine (i.e. L-2-amino-4-(guanidinooxy)butyric acid) is a potent L-arginine antagonist that has documented anti-cancer (Thomas *et al.*, 1987; Rosenthal, 1998; Smith *et al.*, 2003; Ghogomu, 2002, Bence *et al*., 2002, Bence *et al*., 2003), apoptosis (Chinkwo *et al.*, 2003, Jang *et al., 2002*,) as well as anti-infective and anti-oxidant effects (Katarere & Eloff, 2003). It is also thought to affect regulatory and catalytic reactions of arginine metabolism, arginine uptake, formation of structural components and other cellular processes. Canavanine is a potentially deleterious arginine antimetabolite and has been linked to an induction of autoimmune disease e.g. systemic lupus erythematosus (Brown et al. 2001, Farnsworth 1995), but its adverse effects are most likely to occur with very high levels of the compound and /or in presence of low arginine levels and/or in certain predisposed individuals. The level of canavanine in presently used *S. frutescens* preparations is expected to be quite low (around 2.5mg per gram dry powder), but its bioavailability, pharmacokinetics, possible accumulation and effect during chronic use of the plant has not yet been established. Similarly, this type of information is also not available for the other possible active constituents of this plant medicine.

To our knowledge no clinical study has yet been done on *S. frutescens.* However a pre-clinical study in vervet monkeys (*Chlorocebus aethiops*) was recently done at the Medical Research Council in Tygerberg, South Africa ([**www.sahealthinfo.org/traditionalmeds/firststudy.htm**](http://www.sahealthinfo.org/traditionalmeds/firststudy.htm)**,** appended). The monkeys were given as much as 9 times the recommended dose (viz. 81.0mg/kg per day p.o. for 3 months), but (compared to control monkeys not receiving the plant material) no clinically significant changes were seen in haematology, blood biochemistry, and physiological variables measured. That is, *S. frutescens* was found to be non-toxic in vervet monkeys.

**Note 1:** While the name *Sutherlandia frutescens* (L.) R.Br. is the adopted scientific name for the plant species referred to in this project, the taxonomy of this species has not been resolved and several names for this taxon are currently in use. This will not impact this study as plant material of this taxon is to be obtained from a reputable commercial supplier, and is from the same chemotype used for the animal toxicity studies. Research on resolving the taxonomy and nomenclature of this taxon could be included in a future project of TICIPS. For the purposes of this Pilot Project the taxa named *Sutherlandia frutescens* (L.) R.Br., *Sutherlandia microphylla* Burch. ex DC.,

*Sutherlandia frutescens* subspecies *microphylla* (Burch. ex DC.) Moshe & van Wyk *ined.* and *Lessertia frutescens* (L.) P.Goldblatt & J.C.Manning are considered to be taxonomically identical.

**1.2 Study Rationale**

There has been few properly conducted clinical trials done on traditionally used herbal medicines in South Africa and *S. frutescens* is no exception. Nevertheless this herbal medicine is being used widely without any apparent recorded adverse effects and has the potential to be of immense benefit. However, it contains some constituents that may not only produce beneficial, but also adverse effects. Its safety needs to be scientifically established, particularly under the circumstances and conditions (i.e. chronic use) in which it is being used.

Given the widespread indigenous use of *S. frutescens* as a phytotherapeutic, and its non-toxic effects shown in the pre-clinical studies in a primate, it is logical to now assess its safety, during chronic use, in a phase 1 clinical trial. Only thereafter could the evaluation of its clinical effects be considered.

Many herbal medicines such as S*utherlandia* are increasingly being commercially exploited and advocated for use still based only on unproven claims of efficacy and safety arising from poorly monitored (sometimes even dubious) African traditional medicine use practices. To derive optimal benefit from herbal medicines both their preparation and use (whether in traditional setting and/or along with non-traditional medicine) should be thoroughly evaluated and standardized. However, this is not receiving adequate attention in South Africa at present. Therefore, the development of added capacity and expertise to do such clinical evaluation of traditionally used herbal medicines at UWC would be a most worthy endeavor (and an additional objective with this study).

##### STUDY OBJECTIVES

The primary objective of this study is to investigate the safety of *S. frutescens* subsp. *microphylla* capsules in healthy adult volunteers by assessing liver, kidney, muscle, metabolic, intestinal, cardiac and bone marrow function, as well as physiological and physical status.

A secondary objective is to determine whether any of the potential active constituents (esp. canavanine) of the plant is present in the blood of healthy adult volunteers chronically taking capsules containing *S. frutescens* subsp. *Microphylla* leaf powder.

In addition records of daily study medication use and returned medication will be used to measure and monitor subject compliance in taking such plant medicines.

##### STUDY PLAN AND PROCEDURES

##### Study design

This is a three months long, randomized, double blind, placebo controlled study to evaluate the safety of *S. frutescens* (subsp. *Microphylla)*. Subjects must be healthy consenting adult volunteers who are not taking any other medication and comply with the inclusion and exclusion criteria listed below. Complying subjects will be randomized to receive capsules containing either 400mg of *S. frutescens* subsp. *microphylla* per or placebo material of similar appearance (i.e. lactose and starch plus small amount of dried powdered spinach). Subjects will take 1 capsule twice a day for 3 months. The subjects will come for 5 visits during the study. Visit 1 and 2 must occur within 14 days of each other, all the other visits will be four weeks (± 3days) apart and the time for visits 4 and 5 scheduled within ninety minutes from the time of visit 3. Volunteers will be screened at visit 1, and if they meet the inclusion/exclusion criteria randomized for inclusion at visit 2.

At the latter visit (and visits 3 & 4) they will be issued with 1 month’s supply of active or placebo capsules and a diary card and instructed to commence (or continue) with the taking of the respective preparations. At each visit (except visit 2) safety assessments will be done and blood samples drawn, always before the ingesting of the first dose of preparation for that day.

The study will be conducted at Tijger Trial Center situated in Bellville, South Africa.

**3.1.1 Scheduled clinic visits**

| **Visit number** | **Type of visit** | **Day number (& days between visits)** |
| --- | --- | --- |
| Visit 1 | Information, Screening & Consent | Day –7 |
| Visit 2 | Randomization & 1^st^ Drug issue visit | Day 1 (28 ± 3 days before Visit 2) |
| Visit 3 | After 1 month’s treatment | Day 28 (28 ± 3 days after Visit 1) |
| Visit 4 | After 2 month’s treatment | Day 56 (56 ± 3 days after Visit 1) |
| Visit 5 | After 3 month’s treatment | Day 84 (84 ± 3 days after Visit 1) |

### Visits and assessments

| **Week** | **-1** | **0** | **4** | **8** | **12** |
| --- | --- | --- | --- | --- | --- |
| **Visit** | **1** | **2** | **3** | **4** | **5** |
| Informed consent | x |  |  |  |  |
| Medical/ smoking history | x |  |  |  |  |
| Inclusion/Exclusion criteria | x |  |  |  |  |
| Physical examination, Height and weight | x |  | x | x | x |
| Vital signs: heart rate, blood pressure, etc | x |  | x | x | x |
| *Pregnancy test | x |  | x |  | x |
| Allocation of subject number | x |  |  |  |  |
| Laboratory tests: Haematology & biochemistry: | x |  | x | x | X |
| Active constituent levels in blood |  | X | x | x | x |
| Adverse events |  | X | x | x | X |
| ECG | X |  | x | x | X |
| Urine dipstix: | X |  | x | x | X |
| Study medication - Dispensed/Returned |  | D | R/D | R/D | R |
| Diary – Issued/Returned |  | I | R/I | R/I | R |
|  |  |  |  |  |  |

* Females of childbearing age

**3.2 Study population**

The study population will be healthy adult volunteers who will be mainly recruited from the UWC campus. The investigator should complete a subject screening log to document subjects considered for enrolment, but never enrolled to establish that the subject population is selected without bias. Recruitment will continue until 24 subjects are randomized into the 2 groups (test and placebo) of 12 subjects each.

**3.2.1 Inclusion criteria**

To be enrolled, the following criteria have to be fulfilled at Visit 2:

1. Adult of either sex between 18 and 45 years.
2. Body weight within 25% of the appropriate weight range (see appendix 10.4)
3. No significant diseases or clinically significant abnormal laboratory values during screening
4. 12-lead ECG without significant abnormalities
5. On no regular medical treatment
6. Able to communicate effectively with study personnel
7. Able to be informed of the nature of the study and willing to give written informed consent (Consent must be obtained before any study-related procedures are conducted).
   - 1. **Exclusion criteria**

Any of the following is regarded as a criterion for exclusion from the study:

1. Hypersensitivity or idiosyncratic reaction to any drugs or herbal products.
2. Any disease or condition which might compromise the haematopoietic, renal, endocrine, pulmonary, central nervous, cardiovascular, immunological, dermatological, gastrointestinal or any other body system.
3. History of allergic conditions – asthma, urticaria, eczema.
4. History or presence of dyspepsia, gastric ulcer or duodenal ulcer.
5. History of autoimmune disorders e.g. systemic lupus erythematosus, haemolytic anemia.
6. History of psychiatric disorders.
7. Intake of any medication within 14 days before start of the study
8. Subjects who are scheduled to undergo hospitalization for surgery during the study period.
9. Recent history (<2 years) of alcoholism (alcohol abuse) or unlikely to refrain from excessive alcohol consumption during the study period.
10. Smokers, who smoke more than 10 cigarettes per day and cannot refrain from smoking during the study period.
11. Presence of clinically significant abnormal laboratory results during screening.
12. Pregnancy or breastfeeding
13. Females of childbearing age potential not using medically accepted contraceptive measures, as judged by the investigator.
14. Use of any recreational drugs or a history of drug addiction.
15. Participation in a clinical study of any investigational product 1 month prior to visit 1 or during the study.
    - 1. **Justification for inclusion and exclusion criteria**

The criteria are set to minimize the risk to the volunteers, to ensure a subject population that will enable the investigation of the set objectives, to provide equal opportunity for inclusion and not to exclude subjects that may be usual users of the herbal medicine. Because this is the first formal safety study on *Sutherlandia frutescens* children are excluded from it; their inclusion may however be considered in subsequent studies.

- - 1. **Criteria for discontinuation**

Subjects may be discontinued from study treatment and assessments at any time, at the discretion of the investigator. Specific reasons for discontinuing a subject from the study are:

1. Withdrawal of informed consent.
2. Development of exclusion criteria, pregnancy or other safety reasons during the study.
3. Protocol non-compliance.
4. Incorrect enrolment or randomization of the subject.

For subjects withdrawn from the study, the same measurements and assessments should be performed as done at Visit 5. Adverse events should be followed up and diaries and study medication returned by the subjects.

- 1. **Investigational Products and Treatments**

**3.3.1 Treatment Schedule**

At Visit 2, eligible subjects will receive the study drug in a capsule form, either the test product *Sutherlandia frutescens* (subsp. *Microphylla*) or the placebo product.

The subjects will take one capsule of study medication orally with water (ca. 100ml) every morning upon rising (ca. 06:00 to 10:00) and every night before going to bed for 12 weeks. The capsule may be taken with meals.

Everyday the subject will record if and the actual time when each dose of the medicine is taken. Subjects will be supplied with medication once monthly at a further two of their other appointed visits (i.e. visits 3 & 4).

- - 1. **Randomisation**

At Visit 1, subjects who are screened for the study will receive an enrolment code: E – code = E + a consecutive number of 3 digits. Subjects who fulfill all inclusion criteria and meet none of the exclusion criteria will be allocated a subject number: S – code = S + a 2 digit number. The order of receiving the test and placebo product will be in accordance with the code of randomisation. Eligible volunteers, who for whatever reason, do not report to the clinic to receive the first supply of herbal medicine after having been selected for the study will be referred to as discontinuers in the report. Once allocated, the volunteer randomization numbers will be used to identify the volunteer during the remainder of the study. A volunteer who, for whatever reason, withdraws or is withdrawn from the study after having been allocated a subject randomization number, will be classified as a dropout, and identified as such in the relevant Case Report Form (CRF).

- - 1. **Identity of study products**

The study product is a standard (20mm x 6mm) capsule filled with Sutherlandia leaf powder. Before use, the consistency of the product will be validated by pharmaceutically evaluating samples of the capsules for mass and content uniformity and dissolution profile (using canavanine and/or pinitol as markers). The product will then be repacked (in quantities of 60) into containers identical to that to be used for the placebo capsules. Identically looking placebo product will be prepared by filling the capsules with a mixture comprising appropriate amounts of lactose, starch and a small amount of dried spinach leaf powder. The placebo products and test product validation will be done in the Pharmaceutics Laboratory, School of Pharmacy, UWC. Assaying for the canavanine and pinitol will be done (at the MRC) using a validated LCMS assay based on the method of Petritis *et al* (2000) and a gas chromatographic method based on the method of Lein *etal*. (2002), respectively. A certificate of analysis will be available before the test product is used.

Each monthly supply of capsules supplied in sealed plastic containers will be marked with the following label:

Study code: TICIPS001 Visit no:

Investigator: Dr Subject no:

Investigational product: *Sutherlandia frutescens* or placebo

Dosage form: 1 capsule twice daily

**Store below 30°C Exp Date:**

**For clinical study use only**

**3.3.4. Storage and Accountability**

All study drugs must be kept in a secure place under adequate storage conditions – protected from moisture and light. Records of dispensing and returns will be maintained by the trial center (TTC). The subject must return all unused study medication for each treatment period to the trial center.

- - 1. **Allowed medication**

Preferably no other medicine should be taken by the subjects. The use of any incidental medication (e.g. mild analgesics, oral contraceptives, etc) must be recorded.

- - 1. **Compliance**

The patient will record the use of study medication in a diary on a daily basis and the adherence to the prescribed treatment will be checked at every clinic visit.

# STUDY MEASUREMENTS AND ENDPOINTS

#### In this study the following endpoints will be measured/recorded

- 1. **Primary Safety Endpoints.**

1. Adverse events (type and frequency) see
2. Physical examination parameters (body mass, height, etc)
3. Vital signs: blood pressure, heart rate, respiratory rate, body temperature
4. ECG
5. Urine dipstix: leucocytes, nitrate, urobilinogen, protein, pH, blood, specific gravity, ketones, bilirubin and glucose
6. Laboratory tests

Haematology: RBC, HB. MCHC, MCH, MCV, RDW, Hct, Hb, WBC Differential count –lymphocytes monocytes, neutrophils, eosinophils, basophils, CD4+, CD8+.

Biochemistry: Serum - Bilirubin (total and conjugated) AST, ALT, ALP, GGT, LDH, CK, Protein, albumin, globulin, cholesterol (total, LDL-C, HDL-C), glucose urea, creatinine, sodium, potassium, chloride, calcium, magnesium.

- 1. **Secondary Safety Endpoints**

Plasma levels of active constituent levels: Canavanine, (and GABA and/or pinitol).

- 1. **Measurements at each visit.**

See section 3.1.1 for the visit schedule.

**Visit 1: Screening Visit**

The subjects will be examined within 14 days prior to the first ingestion of the investigational products to assess their eligibility to participate. Each subject will consent in writing (Appendix 10.2) to the screening process before the start of the examination. The consent form will also include the study information leaflet.

The examinations and investigations will include:

- Medical history, including history of past use of medications, demographics (date of birth, sex, race) and alcohol and tobacco consumption patterns.
- Physical examination including assessment of general appearance, cardiovascular, lungs, mouth, throat and abdomen and measurement of height and body weight. The examination will be made in accordance with the normal clinical routines at the trial center.
- Vital signs: systolic and diastolic blood pressure in supine (after 5 minutes rest) and standing positions (1minute), pulse, respiratory rate and oral temperature.
- ECG: standard 12-lead (see section 4.4.2).
- Laboratory tests: Haematology, serum biochemistry and urine analysis (see section 4.4.3.2).
- Urine β-HCG pregnancy test in non-hysterctomized volunteers. Fertile females must have a negative pregnancy test.
- Baseline plasma levels of canavanine (and GABA and/or pinitol). (see section 4.4.3.3)

**Visit 2: Day 1 - Randomization & 1^st^ Drug Issue**

- Volunteers will be seen at the Trial Center and investigator will confirm that volunteers are healthy and still eligible for inclusion in the study.
- The pre-dose safety assessments and eligibility checks will be recorded on the relevant pages in the individual CRFs.
- The randomized subject will be issued with 1 month’s supply of trial medication, explained how to take the medication and given next appointment date. Subject should start the first dose at the recommend time (ca: 06:00 – 10:00) on the day of drug issue or start the next day.
- The randomized subject will also be issued with a diary and instructed on how to complete it.

**Visits 3 to 5: Treatment and assessment**

For these visits the examinations and investigations will include:

- Collection of returned trial medication and diary cards and debriefing on information in diary card (esp. w.r.t. incidence and type of recorded adverse events).
- Physical examination (as for visit 1)
- Vital signs: systolic and diastolic blood pressure in supine (after 5 minutes rest) and standing positions (1minute), pulse, respiratory rate and oral temperature.
- ECG: standard 12-lead (see section 4.4.2).
- Laboratory tests. Haematology, serum biochemistry and urine analysis (see section 4.4.3.2).
- Urine β-HCG pregnancy test in non-hysterectomized volunteers at (visit 3 and 5 only). Fertile females must have a negative pregnancy test.
- Baseline plasma levels of canavanine (and GABA and/or pinitol). (see section 4.4.3.3)
- Except for visit 5, new issue of trial medication and diary card.

**Note:** For any subject in which any of the above tests indicated levels outside the normal range, a follow-up visit (visit 6) will be scheduled for an appropriate time (1 or 3 months post visit 5). All or the above tests will be repeated at this visit.

- 1. **Specific detail on measurements**

**4.4.1 Adverse events**

An adverse event is the development of an undesirable medical condition - e.g. symptoms or abnormal results of an investigation - or the deterioration of a pre-existing medical condition (not relevant in this study). AE’s will be collected by means of a standard question: “Have you had any health problems since the previous visit?” AE’s will be recorded at every visit. Spontaneously reported AE’s and/or observed AE’s and the subject’s response to this question will be recorded on the AE form with information about seriousness, action taken, date of onset and recovery, maximum intensity and outcome. The subjects will be asked to assess the intensity of the reported Adverse Event according to the following scale:

Mild = awareness of sign or symptom, but easily tolerated

Moderate = discomfort sufficient to cause interference with normal activities

Severe = incapacitating, with inability to perform normal activities.

A Serious Adverse Event is an adverse event occurring during any phase of the study and at any dose of the investigational product or placebo, which fulfils one or more of the following criteria:

- Results in death;
- Is immediately life-threatening;
- Requires in-subject hospitalization;
- Results in persistent or significant disability or incapacity.

The causality of Serious Adverse Events (i.e. the relationship to study treatment) will be assessed by the investigators, who in completing the relevant Case Report Form must answer ‘yes’ or ‘no’ to the question “Do you consider that there is a reasonable possibility that the event may have been caused by the study medication?” The following factors should be considered when deciding if there is a “reasonable possibility” that an Adverse Event may have been caused by the investigational product.

- Time course of events and exposure to suspect drug – did the AE occur in a reasonable temporal relationship to the administration of suspect drug?
- Dechallenge experience – did the AE resolve or improve on stopping or reducing the dose of the suspect drug?
- Rechallenge experience - did the AE reoccur if the suspected drug was reintroduced after having stopped?
- Laboratory tests – has a specific laboratory investigation confirm the relationship?
- No alternative cause - the AE cannot be reasonably explained by another aetiology such as an underlying disease (not previously present), other drugs or environmental factors.

There would not be a “reasonable possibility” of causality if none of the above criteria apply or where there is evidence of exposure and a reasonable time course, but any dechallenge is negative or there is another more likely cause of the AE.

In this study the Adverse Events will be noted from the interview at the time of visit and from the daily input in the diary.

- - 1. **12- Lead ECG**

Standard 12-lead ECGs will be performed at screening and at visits 3 to 5 (and post-study follow-up visit). Volunteers will only be randomized if the ECG is normal, or any abnormalities that are noted are considered by the investigator to be not clinically relevant.

- - 1. **Laboratory Investigations**

Blood and urine will be collected for clinical pathology tests at the screening visit, at visits 3 to 5, and, if required, at any post-study safety follow-up visits (or upon withdrawal). The following variables will be measured.

**4.4.3.1. Haematology:** (10ml blood sample)

**Red cells:** Erythrocytes, Haemaglobin. Haematocrit, MCV, MCH and MCHC.

**White cells:** Leucocytes and 5 part differential (neutrophils, lymphocytes. monocytes, eosinophils and basophils); CD4+ and CD8+ counts.

**Platelets**

**4.4.3.2 Clinical Chemistry:** (2 x 10ml blood samples)

**Serum analysis:** Total bilirubin, ALP, GGT. ALT, AST, LDH,CK, TSH/T3/T4, cholesterol (total, LDL-C, HDL-C);

**Serum electrolytes:** Sodium, potassium, chloride, calcium, magnesium, phosphate, urea, creatinine

**Serum proteins:** Total protein, albumin, globulin

**Plasma glucose** (non-fasting)

**Urinalysis** (Multistix^R^): SG, pH, protein, glucose, ketone, bilirubin, blood and urobilinogen.

**Urine β-HCG pregnancy test**

The Pathcare^R^ Pathology Laboratory, Cape Town, SA will perform all the laboratory investigations. Urinanalysis will be performed at the trail site. The samples will be processed and shipped as per instructions of the pathlogy laboratory.

For all laboratory investigations the investigator will receive actual printouts of the data of the subjects from Pathcare^R^ Pathology Laboratory. A signed original printout will be attached to the CRF and a copy thereof will be filed in the subject file. If the copy seen by the investigator is the faxed copy, then this is the copy that must be signed and retained. The reference ranges will be provided in the clinical trial manual (investigator’s file) together with the descriptions of the laboratory methods used. The laboratory will provide normal ranges on each report to ease the investigator’s assessment.

As part of the overall safety monitoring plan, the safety monitor (Dr FJ Maritz) will also assess the data, particularly those falling outside the normal range.

- - - 1. **Blood levels of active constituent (1x10 ml)**

At visits 2 to 5 venous blood samples (10 ml each) will be collected into heparinised glass tubes for assay of canavanine , GABA and pinitol concentrations. The blood sample (trough) will always be taken before the first trial medication dose for that day is taken. The blood samples will be collected in tubes labeled with the following information: Study number (TICIPS001); Analytes (canavanine, GABA, pinitol); study day; subject study number and initials; blood sample number (visit 2, 3,4 or 5) and sampling time.

Blood samples will be handled on ice between collection and centrifugation. Within 15 to 30 minutes of collection, the blood samples will be centrifuged at approx. 2600 g and approx 4^o^C for 10 minutes after which the supernatant will be divided into 3 approximate aliquots. The aliquots will be transferred to the appropriately labed glassed tubes (75 x 12 mm silanised borosilicate test tubes with plastic ripple caps. The samples will be stored in a -20 ^o^C freezer pending shipment for assay (to Pharmaceutics Lab, UWC or MRC lab, less than 10 km away). Samples will be kept frozen during the shipment by packing them in “dry ice”.

The plasma samples will be assayed for the targeted plant constituents using high performance liquid chromatography (HPLC) with ultra violet (UV) or mass spectrometric (MS) detection (canavanine) or fluorescence detection (GABA) or gas chromagtography (pinitol) in validated procedures developed from the methods of Petritis *et al* (2000), Shiah *et al* (2003) and Lein *et al*. (2002), respectively. (Note: The exact details of these assay procedures are still being finalized and validated and will be supplied later, before commencement of the study).

- 1. **Measurements recorded daily in a diary**

A diary will be filled in at home during the entire study in the morning after taking the morning dose and at night after the evening before going to bed. The following variables will be recorded:

1. Intake of study medication (if, time and if with food)
2. Occurrence of any adverse effects.
3. Intake of any incidental medication

The subjects will record the time (morning and evening) that the study medication has been taken. The recordings are a measurement of compliance.

1. **STATISTICAL METHODS**

**5.1 Determination of sample size**

In the absence of any useful specific safety data and because the level of safety of the trial medicine, based on the absence of safety incidences reported during the traditional use of Sutherlandia, is expected to low, a convenience sample size of 24 subjects was chosen.

- 1. **Statistical analysis**

A single statistical analysis will be performed at the end of the study. An intention to treat (ITT) approach will followed, i.e. statistical analysis of safety will be based on data from all patients who were randomized and from whom meaningful data were collected. Data will be displayed graphically for visual inspection. Descriptive statistics will be presented as means, SEM and ninety percent confidence levels of the means.

**Baseline characteristics.** The subject disposition will be summarized. Protocol violations will be listed per subject, describing the nature of the violation. Subjects failing to complete the study (as well as the times and reasons for discontinuation) will be displayed. The demographic, background and baseline data will be presented descriptively.

**Analysis of Safety.** Adverse Events, as reported throughout the course of the trial will be listed individually, per treatment group. Pre-, study and post-study findings of **physical examination, vital sign variables, laboratory variables** (haematology, clinical chemistry and urinalysis), and **12-lead ECG** will be listed individually and summarized; values outside the normal range will be listed.

**Analysis of Constituent levels**. The trough levels of active constituents obtained during the treatment will be compared to that found before the start of the taking of trial treatment and the averaged levels obtained in the trial medication and placebo groups, compared and analyzed using an analysis of variance model.

- 1. **Changes to the Clinical Study Protocol**

The Medicines Control Council (MCC) and the Local Ethics Committees (e.g. the Pharmaceutical Trials IRB of University of Stellenbosch; OHRP registration code IRB0000391) will be notified of any amendments to the Clinical Study Protocol and no changes will be effected without approval from the Regulatory Authorities.

1. **ETHICS**

**6.1 Ethics review**

The final study protocol, including the final version of the Subject Information and Consent Forms, must be approved in writing by an Independent Ethics Committee (IEC) (e.g. the Pharmaceutical Trials IRB of University of Stellenbosch) and the MCC before enrolment of any subject into the study. The Principle Investigator (Clinical Trial Manager) is responsible for informing the IEC of any serious adverse events (SAE) and amendment to the protocol as per regulatory requirement.

**6.2. Ethical conduct of the study**

The study will be performed in accordance with the ethical principles in the Declaration of Helsinki (see Appendix 10.1), and that are consistent with Good Clinical Practice and applicable regulatory requirements. Insurance cover will be taken out by TICIPS to cover

- 1. **Subject information and consent**

The Investigator will ensure that the subject is given full and adequate oral and written information about the nature, purpose, possible risk and benefit of the study. Subjects must also be notified that they are free to discontinue from the study at any time. The subject should be given the opportunity to ask questions and allowed time to consider the information provided. The subject’s signed and dated informed consent must be obtained before conducting any study specific procedure. The investigator must store the original, signed Subject Informed Consent Form and a copy must be given to the subject. Samples of the English version of the Subject Information and Consent Forms are enclosed (Appendix 10.2). Afrikaans and Xhosa translations of the approved English version will also be provided and submitted to IEC.

- 1. **Subject data protection**

The Subject Information and Consent Form will explain that study data will be stored in a computer database, maintaining confidentiality. Subjects in this database will be identified by initials or enrolment code / subject number only. Authorized representative of a regulatory authority (e.g. MCC) may require direct access to parts of the trial site records relevant to the study, including subjects’ medical history for data verification purposes.

The Investigator must keep a Subject Identification List of all subjects that have signed the informed consent, including subject number, full name and last known address.

**6.5 Insurance and Indemnity**

All subjects participating in this trial will be covered for any trial medicine related adverse effects through insurance cover to be taken out by TICIPS.

**7. DATA QUALITY ASSURANCE**

Data from the study will be collected in CRFs. Data editing will be performed at the trial center, comparing source and CRF entries. Data will be entered in a blind mode.

During the study an independent monitor will visit the investigational site to confirm that the facilities remain acceptable, that the investigational team is adhering to the protocol and that data are being accurately recorded in the CRFs. Source data verification (a comparison of the data in the CRF with the subjects laboratory test results and other source documents) will also be performed.

Authorized representatives of the regulatory authority (e.g. MCC) may visit the center to perform inspections, including source data verification.

Clean File for the final database will be declared when all data have been entered and a quality check on a sample of the data has been performed. The database will be locked after Clean File has been declared and data extracted for statistical analysis. Treatment code will not be broken until clean file.

Study committee meetings will be held as needed prior to or during the study. The medical, nursing and other staff involved in the study will receive proper education/information on how to conduct the study according to the protocol.

**8. STUDY TIME TABLE AND TERMINATION**

First subject in March 2004

Last patient out July 2004

Clean File August 2004

Study Report September 2004

**9. REFERENCES**

1. Van Wyk, B. & Gericke, N. (2000). People’s Plants, Briza Publications, SA.
2. Van Wyk, B., Van Oudtshoorn, B. and Gericke, N. (2000). Medicinal Plants of South Africa, 2^nd^ Ed, Briza Publications, SA.
3. [**www.astral-natural.com/Africana.htm**](http://www.astral-natural.com/Africana.htm) (2001). Ethnomedicine Report of Sutherlandia frutescens. The International Conference on Traditional Medicine in HIV/AIDS and Malaria, Abuja. Nigeria.
4. Davis A., Christiansen M, Horowitz JF, Klein S, Hellerstein MK, and Ostlund RE Jr. (2000). Diabetes Care, 23(7), 1000 -1005.
5. Rosenthal, G., In: Frontiers and New Horizons in Amino Acid Research, K Takai, (Ed), Elsevier, 1992.
6. [**www.sutherlandia.org/chemistry.html**](http://www.sutherlandia.org/chemistry.html)**.** (2001). Chemistry and Pharmacology of the Sutherlandia frutescens.
7. Thomas, D.A. and Rosenthal, G.A. (1987). Toxicity and pharmacokinetics of the non-protein amino acid L-canavanine in the rat. Toxicol Appl Pharmacology, 91:395-405,1987.
8. Rosenthal, G.A. (1998). L-canavanine: A potential chemotherapeutic agent for human pancreatic cancer. Pharmaceutical Biology: 36:194-201.
9. Smith, D., de Kock, M., Seier, J., and Johnson Q., (2003) The effect of Sutherlandia frutescens extracts on pathogenic microbes, cancer cells and primate health. 2003: BS (Honours) project, UWC.
10. Ghogomu, T.A. (2002). Evaluation of Sutherlandia frutescens for anti-cancer activity. MS thesis, UWC.
11. Chinkwo, K.A., Meyer, J.J.M., Scott, G. and Rees, D.J.G., (2003). Bioactivity guided fractionation of Sutherlandia frutescens for the isolation of compounds, which induces apoptosis. Paper presentation at the SAAB and ISE Conference, Pretoria – South Africa, 7 – 11 January 2003.
12. Singh R.K., Pandey B.L., Tripathi, M. and Pandey,V.B. (2001). Anti-inflammatory effect of (+)-pinitol. Fitoterapia: 72(2); 168-170.
13. Farnsworth N. R., ( 1995). Alfalfa pills and autoimmune diseases. Am J Clin Nutr: 62(5); 1026-1028.
14. Katerere, D.R.P. and Eloff, J.N., (2003). Antibacterial and antioxidant activity of Sutherlandia frutescens*.* Paper presentation at the SAAB and ISE Conference, Pretoria – South Africa, 7 – 11 January 2003.
15. [**www.sahealthinfo.org/traditionalmeds/firststudy.htm**](http://www.sahealthinfo.org/traditionalmeds/firststudy.htm)**.** (2002). A toxicity study of Sutherlandia leaf powder (Sutherlandia frutescens, sub-species microphylla) consumption. Medical Research Council, Tygerberg, South Africa.
16. Larner, J., Allan G., Kessler, C., Reamer, P., Gunn, R. and Huang, L.C. (1998) Phosphoinositol g;ycan-derived mediators and insulin resistance. Prospects for diagnosis and therapy. Journal of Basic and Clinical Physiology and Pharmacology : 9(2-4), 127-137.
17. Bence, A. K., Worthen, D.R., Adams, V.R. and Crooks, P.A. (2002). The antiproliferative and immunotoxic effects of l-canavanine and L-canaline. Anti-Cancer Drugs: 13(3), 313-320.
18. Brown, D.L., Naiki, M. and Gerswhin, M.E., (2001). Does L-canavanine ingestion induce murine SLE? Paradoxical effects on survival of BALB/c mice. Journal of Nutritional Immunology: 5(3/4); 17-27.
19. Montanaro, A. and Bardana E.J. Jr., (1991). Dietary amino acid-induced systemic lupus erythematosus. Rheum Dis Clin North Am: 17 (2); 323-332.
20. Jang, M.H., Hun, D.Y., Rue, S.W., Han, K.H., Park, W. and Kim, Y.H. (2002). Arginine antimetabolite L-canavanine induces apoptotic cell death in human Jurkat T cells via caspase-3 activation regulated by Bcl-2 or Bcl-xL. Biochemical and Biophysical research Communication: 295(2); 283-288
21. Petritis K., Chaimbault P., Elfakir C. and Dreux, M. (2000). Parameter optimization of underivatized protein amino acids by liquid chromatography and ionspray tandem mass spectrometry. J Chromatography A: 896(1-2); 253-263.
22. Lein S., Van Boven M., Holser R., Decuypere E., Flo G., Lievens S. and Cokelaere M. (2002). Simultaneous determination of carbohydrates and simmondsins in jojoba seed meal (Simmondsia chenesis) by gas chromatograpy. J Chromatograpy: 977(2); 257- 264.
23. Shiah I-S., Tatham L. N., Gau Y-C. and Baker G. B (2003). Effect of lamotrigine on plasma GABA levels in healthy humans. Progress in Neuro-Psychopharmacology & Biological Psychiatry: 27; 419 –423.

###### APPENDIX 10.1

DECLARATION OF HELSINKI

| **WORLD MEDICAL ASSOCIATION DECLARATION OF HELSINKI Ethical Principles for Medical Research Involving Human Subjects** |  |
| --- | --- |

Adopted by the 18th WMA General Assembly, Helsinki, Finland, June 1964, and amended by the
29th WMA General Assembly, Tokyo, Japan, October 1975
35th WMA General Assembly, Venice, Italy, October 1983
41st WMA General Assembly, Hong Kong, September 1989
48th WMA General Assembly, Somerset West, Republic of South Africa, October 1996
and the 52nd WMA General Assembly, Edinburgh, Scotland, October 2000
Note of Clarification on Paragraph 29 added by the WMA General Assembly, Washington 2002

**INTRODUCTION**

- 1. The World Medical Association has developed the Declaration of Helsinki as a statement of ethical principles to provide guidance to physicians and other participants in medical research involving human subjects. Medical research involving human subjects includes research on identifiable human material or identifiable data.
  2. It is the duty of the physician to promote and safeguard the health of the people. The physician's knowledge and conscience are dedicated to the fulfillment of this duty.
  3. The Declaration of Geneva of the World Medical Association binds the physician with the words, "The health of my patient will be my first consideration," and the International Code of Medical Ethics declares that, "A physician shall act only in the patient's interest when providing medical care which might have the effect of weakening the physical and mental condition of the patient."
  4. Medical progress is based on research, which ultimately must rest in part on experimentation involving human subjects.
  5. In medical research on human subjects, considerations related to the well-being of the human subject should take precedence over the interests of science and society.
  6. The primary purpose of medical research involving human subjects is to improve prophylactic, diagnostic and therapeutic procedures and the understanding of the aetiology and pathogenesis of disease. Even the best-proven prophylactic, diagnostic, and therapeutic methods must continuously be challenged through research for their effectiveness, efficiency, accessibility and quality.
  7. In current medical practice and in medical research, most prophylactic, diagnostic and therapeutic procedures involve risks and burdens.
  8. Medical research is subject to ethical standards that promote respect for all human beings and protect their health and rights. Some research populations are vulnerable and need special protection. The particular needs of the economically and medically disadvantaged must be recognized. Special attention is also required for those who cannot give or refuse consent for themselves, for those who may be subject to giving consent under duress, for those who will not benefit personally from the research and for those for whom the research is combined with care.
  9. Research Investigators should be aware of the ethical, legal and regulatory requirements for research on human subjects in their own countries as well as applicable international requirements. No national ethical, legal or regulatory requirement should be allowed to reduce or eliminate any of the protections for human subjects set forth in this Declaration.

1. **BASIC PRINCIPLES FOR ALL MEDICAL RESEARCH**
   1. It is the duty of the physician in medical research to protect the life, health, privacy, and dignity of the human subject.
   2. Medical research involving human subjects must conform to generally accepted scientific principles, be based on a thorough knowledge of the scientific literature, other relevant sources of information, and on adequate laboratory and, where appropriate, animal experimentation.
   3. Appropriate caution must be exercised in the conduct of research, which may affect the environment, and the welfare of animals used for research must be respected.
   4. The design and performance of each experimental procedure involving human subjects should be clearly formulated in an experimental protocol. This protocol should be submitted for consideration, comment, guidance, and where appropriate, approval to a specially appointed ethical review committee, which must be independent of the investigator, the sponsor or any other kind of undue influence. This independent committee should be in conformity with the laws and regulations of the country in which the research experiment is performed. The committee has the right to monitor ongoing trials. The researcher has the obligation to provide monitoring information to the committee, especially any serious adverse events. The researcher should also submit to the committee, for review, information regarding funding, sponsors, institutional affiliations, other potential conflicts of interest and incentives for subjects.
   5. The research protocol should always contain a statement of the ethical considerations involved and should indicate that there is compliance with the principles enunciated in this Declaration.
   6. Medical research involving human subjects should be conducted only by scientifically qualified persons and under the supervision of a clinically competent medical person. The responsibility for the human subject must always rest with a medically qualified person and never rest on the subject of the research, even though the subject has given consent.
   7. Every medical research project involving human subjects should be preceded by careful assessment of predictable risks and burdens in comparison with foreseeable benefits to the subject or to others. This does not preclude the participation of healthy volunteers in medical research. The design of all studies should be publicly available.
   8. Physicians should abstain from engaging in research projects involving human subjects unless they are confident that the risks involved have been adequately assessed and can be satisfactorily managed. Physicians should cease any investigation if the risks are found to outweigh the potential benefits or if there is conclusive proof of positive and beneficial results.
   9. Medical research involving human subjects should only be conducted if the importance of the objective outweighs the inherent risks and burdens to the subject. This is especially important when the human subjects are healthy volunteers.
   10. Medical research is only justified if there is a reasonable likelihood that the populations in which the research is carried out stand to benefit from the results of the research.
   11. The subjects must be volunteers and informed participants in the research project.
   12. The right of research subjects to safeguard their integrity must always be respected. Every precaution should be taken to respect the privacy of the subject, the confidentiality of the patient's information and to minimize the impact of the study on the subject's physical and mental integrity and on the personality of the subject.
   13. In any research on human beings, each potential subject must be adequately informed of the aims, methods, sources of funding, any possible conflicts of interest, institutional affiliations of the researcher, the anticipated benefits and potential risks of the study and the discomfort it may entail. The subject should be informed of the right to abstain from participation in the study or to withdraw consent to participate at any time without reprisal. After ensuring that the subject has understood the information, the physician should then obtain the subject's freely given informed consent, preferably in writing. If the consent cannot be obtained in writing, the non-written consent must be formally documented and witnessed.
   14. When obtaining informed consent for the research project the physician should be particularly cautious if the subject is in a dependent relationship with the physician or may consent under duress. In that case the informed consent should be obtained by a well-informed physician who is not engaged in the investigation and who is completely independent of this relationship.
   15. For a research subject who is legally incompetent, physically or mentally incapable of giving consent or is a legally incompetent minor, the investigator must obtain informed consent from the legally authorized representative in accordance with applicable law. These groups should not be included in research unless the research is necessary to promote the health of the population represented and this research cannot instead be performed on legally competent persons.
   16. When a subject deemed legally incompetent, such as a minor child, is able to give assent to decisions about participation in research, the investigator must obtain that assent in addition to the consent of the legally authorized representative.
   17. Research on individuals from whom it is not possible to obtain consent, including proxy or advance consent, should be done only if the physical/mental condition that prevents obtaining informed consent is a necessary characteristic of the research population. The specific reasons for involving research subjects with a condition that renders them unable to give informed consent should be stated in the experimental protocol for consideration and approval of the review committee. The protocol should state that consent to remain in the research should be obtained as soon as possible from the individual or a legally authorized surrogate.
   18. Both authors and publishers have ethical obligations. In publication of the results of research, the investigators are obliged to preserve the accuracy of the results. Negative as well as positive results should be published or otherwise publicly available. Sources of funding, institutional affiliations and any possible conflicts of interest should be declared in the publication. Reports of experimentation not in accordance with the principles laid down in this Declaration should not be accepted for publication.
2. **ADDITIONAL PRINCIPLES FOR MEDICAL RESEARCH COMBINED WITH MEDICAL CARE**
   1. The physician may combine medical research with medical care, only to the extent that the research is justified by its potential prophylactic, diagnostic or therapeutic value. When medical research is combined with medical care, additional standards apply to protect the patients who are research subjects.
   2. The benefits, risks, burdens and effectiveness of a new method should be tested against those of the best current prophylactic, diagnostic, and therapeutic methods. This does not exclude the use of placebo, or no treatment, in studies where no proven prophylactic, diagnostic or therapeutic method exists. See footnote
   3. At the conclusion of the study, every patient entered into the study should be assured of access to the best-proven prophylactic, diagnostic and therapeutic methods identified by the study.
   4. The physician should fully inform the patient which aspects of the care are related to the research. The refusal of a patient to participate in a study must never interfere with the patient-physician relationship.
   5. In the treatment of a patient, where proven prophylactic, diagnostic and therapeutic methods do not exist or have been ineffective, the physician, with informed consent from the patient, must be free to use unproven or new prophylactic, diagnostic and therapeutic measures, if in the physician's judgement it offers hope of saving life, re-establishing health or alleviating suffering. Where possible, these measures should be made the object of research, designed to evaluate their safety and efficacy. In all cases, new information should be recorded and, where appropriate, published. The other relevant guidelines of this Declaration should be followed.

**FOOTNOTE:
NOTE OF CLARIFICATION ON PARAGRAPH 29 of the WMA DECLARATION OF HELSINKI**

The WMA hereby reaffirms its position that extreme care must be taken in making use of a placebo-controlled trial and that in general this methodology should only be used in the absence of existing proven therapy. However, a placebo-controlled trial may be ethically acceptable, even if proven therapy is available, under the following circumstances:

- Where for compelling and scientifically sound methodological reasons its use is necessary to determine the efficacy or safety of a prophylactic, diagnostic or therapeutic method; or

- Where a prophylactic, diagnostic or therapeutic method is being investigated for a minor condition and the patients who receive placebo will not be subject to any additional risk of serious or irreversible harm.

All other provisions of the Declaration of Helsinki must be adhered to, especially the need for appropriate ethical and scientific review.

The Declaration of Helsinki (Document 17.C) is an official policy document of the World Medical Association, the global representative body for physicians. It was first adopted in 1964 (Helsinki, Finland) and revised in 1975 (Tokyo, Japan), 1983 (Venice, Italy), 1989 (Hong Kong), 1996 (Somerset-West, South Africa) and 2000 (Edinburgh, Scotland). Note of clarification on Paragraph 29 added by the WMA General Assembly, Washington 2002.

06.10.2002 09h00

**APPENDIX 10.2a**

**PATIENT INFORMATION LEAFLET AND INFORMED CONSENT**

# PATIENT INFORMATION SHEET AND

# INFORMED CONSENT FORM

**Patient identification number: . . . . / . . . . . Patient initials:**

**Title of the study: A randomized double blind placebo controlled study to investigate the safety of *Sutherlandia frutescens (subspecies microphylla)* in healthy adult volunteers**.

**Protocol number:** TICIPS001

**Sponsor:** The International Centre for Indigenous Phytotherapy Studies (TICIPS)

**Name of Principal Investigator:** Dr H Nell

**Name of Project Leader:** Prof J A Syce

**Address of site/institution where study will be conducted:** Tijger Trial Centre (TTC), First Floor Ward 1A, Karl Bremer Hospital, Bellville, Cape Town, South Africa & UWC, Student Health Centre, Bellville, Cape Town, South Africa).

**INTRODUCTION**

You are being invited to take part in a clinical research study sponsored by **The International Centre for Indigenous Phytotherapy Studies (TICIPS).** TICIPS is made up of staff from the University of the Western Cape, The University of Missouri – Columbia, and the Missouri Botanical Garden in the United State of America who are engaged in joint research on indigenous African plant medicines and nutrients. Before deciding to take part in the study, it is important for you to understand why the research is done and what will happen to you. This information sheet will provide you with information about this study and your rights as a research subject so that you can decide if you want to take part. Please take the time to read this information carefully and ask the investigator when anything is not clear, or if you would like more information*.*

What is the purpose of this study?

The purpose of this study is to determine the safety of the popular plant medicine ***Sutherlandia frutescens*.** The latter is the study drug, a term that may also be used later on in this document or study. Sutherlandia frutescens is also known as Cancer bush in English, Kankerbos in Afrikaans, Unwele in Zulu, etc. We need to accurately establish its safety and to do so first in healthy subjects. That is thus the aim of this study.

In this study *Sutherlandia frutescens (subsp microphyllin*) in capsule form or a similar looking capsule without the plant (i.e. a placebo) will be given to 24 healthy volunteers.

They will take the medicines orally twice daily for 3 months while being monitored for any effects of this plant medicine. The study will be done at Tijger Trial Centre located at Karl Bremer Hospital.

**Do I have to participate in the study?**

Participation in this study is entirely voluntary. It is up to you to decide whether to take part or not.

If you do not want to take part in this study or if you wish to withdraw from the study at any time you may do so without giving a reason and you will not lose any benefits to which you would otherwise be entitled.

If you withdraw from the study the data collected up to the point of withdrawal will be analyzed for the purpose of the study.

If you decide to participate, you will be given this information sheet to keep and be asked to provide your signature indicating your consent.

**Who is eligible to participate in the study?**

About 24 people (older than 18 years and younger than 45) will take part in this study. In order to participate in this trial you need to be in the appropriate weight range, not on any regular medicine, be able to communicate effectively with the study staff and generally be in good health. Apart from being a healthy adult, there are also some other rules for taking part in the study that the study doctor will check by asking you questions and performing a physical examination on you.

You may however not take part in this trial if you are unable to stop smoking for the duration of the trial, are breastfeeding or pregnant, are addicted to drugs or have a past history of drug addiction, or have been involved in any trial within one month of your first visit in this trial.

There is not enough information on the use of *Sutherlandia frutescens* (study drug**)** in pregnant women and the risk to the human unborn baby is unknown. Active constituents in *Sutherlandia frutescens* may cross the placenta and cause harm or increase the risk of deformity in the human unborn baby. Active ingredients from *Sutherlandia frutescens* may also be absorbed in breast milk. Therefore, women who are pregnant, planning to become pregnant, or who are breastfeeding their children are requested not to take part.

**What will happen to me if I participate?**

If you decide to take part you must sign the consent form and will then first be examined to see if you meet all the requirements for participation in the study. These examinations and investigations (at visit 1) will include the taking of a medical history history, including history of past use of medications; a physical examination; the withdrawal of 40ml (approximately 8 teaspoons) of blood for laboratory tests; the recording of an ECG (i.e. an electrocardiogram or recording to check the activity of the heart) and, if you are female, the collection of urine for a pregnancy test.

If you qualify for inclusion you will thereafter, within 14 days of the first examination, be issued (at visit 2) with study medication that you must take as follows: 1 capsule each in the morning and evening with or after food. There will however be 2 study medications, *Sutherlandia frutescens* and placebo capsules, which will be identical in appearance and, you may receive one or the other. The allocation of the capsules is done by random selection and neither you nor the study doctor will know on which capsule you are until the study is completed. You have a one in two chance of being given the study drug or placebo. At this visit (visit 2) you will be issued with a month’s supply of medication as well as a diary record card that you must fill in daily and return, along with unused medication, after 1 month. In the diary card you will record your daily study drug use and if you experienced any adverse effects

You will have to take the study medication for a total of 3 months and return for visits to the trial center every 4 weeks for a further 3 visits. This means that the study will last between 3 and 4 months.

During the subsequent visits (**visits 3 to 5**)*,* you will again be examined by the study doctor and asked you if you have experienced any medical problems since the last visit. The same examinations and investigations done during visit 1(i.e. the recording of an ECG, withdrawal of 40 ml (8 teaspoons) of blood, etc) will be done. You will not receive any medication at the last visit (**visit 5**) when the study ends.

**What do I have to do?**

As a subject in this study, you are responsible for:

- keeping all clinic appointments;
- taking the study medication as explained by the study doctor;
- completing the diary cards that you will receive;
- returning the filled-out cards at each visit;
- telling your study doctor before taking any other medication;
- telling your study doctor as soon as possible if there is a change in your health;

● ensuring, if you are a woman of childbearing age, that you take all the needed precautions not to

fall pregnant while on this study; and

● returning all used and unused medication and the packaging it came in at each visit

If you experience any injuries and need to see a doctor, you must also tell that doctor that you are participating in this research study.

**What risks or discomforts might occur if I participate?**

As with any medication, you may experience some side-effects whist taking the study drug. The aim of this study is indeed to see if *Sutherlandia frutesecens* do indeed cause such adverse effects.

So far it has been reported that the study drug *(Sutherlandia)* only produced occasional incidents of: mild loose stool, mild constipation, dry mouth and, rarely, dizziness in very frail patients.

Several chemical compounds have however been isolated from *Sutherlandia* and these may produce some effects. Most of these would most likely be beneficial and the reason(s) why this plant may be effective in some diseases. But, some of these potential effects (e.g. lowering of blood glucose, enhancing of autoimmune diseases,) may, in some individuals, also be adverse or unwanted. We must be open to the possibility that they can occur. In this study we will specifically monitor the levels of some of these actives substances to see if they may be implicated in any adverse effects of the plant medicine.

Many of the procedures to be used in this study may cause you some discomforts (e.g. the withdrawal of blood may cause slight bruising or possible fainting, there may be discomfort with recording of the ECG, etc), but these will be no more than that experienced in a typical medical examination.

If you are a woman of childbearing potential, you may take part in the study only if you use a medically accepted method of contraception (birth control). ***Medically accepted methods of birth control include*** the use of oral, intramuscular or transcutaneous contraceptive medication, intrauterine devices, surgical methods i.e. hysterectomy or sterilization and, at the discretion of the clinical investigator a few other methods (e.g. barrier methods, vasectomy in regular male partner, etc). The study doctor will discuss this with you at the first visit. In addition, you will have a pregnancy test on your blood at the first visit and each time a blood sample is taken. If the pregnancy test is positive, the study doctor will immediately withdraw your medication.

**What are the possible benefits of participating?**

You may or may not receive any direct benefit from taking the study medication, but all the regular visits and examinations to monitor your health will be provided free of charge. Further, the information we get from this study will extend our existing knowledge and will help to decide if this medication is safe to use in other people such as you.

**Will there be any cost to me if I participate?**

There will be no cost to you for participating in this research study, but you will need to allow enough time for the clinic visits.

You will not be paid for being in this study; all the tests, examinations and study medication will be provided to you at no cost. However, your study doctor will ask you if you had to pay any costs (for example bus, train, taxi fares) in order to come to the clinic. You should keep all receipts of bus, train, taxi fares etc, and give them to your study doctor. You will be reimbursed for all reasonable travel expenses incurred as a result of taking part in the study.

**What if something goes wrong or if I have problems while I am in the study?**

If you experience a study related injury, you will be reimbursed for medical expenses for the treatment of bodily injuries that were caused by the use of the study medication.

An insurance policy that covers any damage to your health arising from your participation in this study has been taken out.

Compensation for a study related injury would be in accordance with established standards set by the Association for British Pharmaceutical Industry (ABPI). The study doctor will give you a copy of the ABPI-guidelines upon your request.

In case of study related injury please contact:

Doctor: Dr Haylene Nell

Contact Numbers: +27 21 918 1399/1272 or 083 448 1456

If you are harmed by your participation in the study, you will be compensated according to the guidelines of the pharmaceutical industry relating to clinical trials (i.e. ABPI guidelines). If you are harmed due to someone’s negligence then you may have grounds for legal action. You are not waiving your legal rights by signing this form.

For further information on the insurance coverage, please ask the study doctor.

**Can I withdraw or be withdrawn from the study?**

Taking part in the study is voluntary. If you decide to take part in the study, you are free to withdraw from the study at any time. If you decide to withdraw from the study, you should inform your study doctor immediately. Your study doctor will not be upset and you will not be penalized in any way, and your future care will not be affected. Should you withdraw from the study, the study data collected before your withdrawal may still be processed along with other data collected as part of the study.

In addition, circumstances may arise that will lead to the ending of your participation in this study. Such circumstances could include:

- medical reasons including pregnancy;
- you not taking your medication or not regularly keeping to the study appointments, or if you do not follow the doctor's instructions;
- you taking a medication that is not allowed in this study;
- if there are not enough patients in the study;
- if the Medicines Control Council or the Committee for Pharmaceutical Trials stops or suspends the investigation.
- If the Sponsor stops the study.

**Will my participation in this study be kept confidential?**

The records that identify you will be kept confidential and, to the extent permitted by the applicable laws and regulations, will not be made publicly available. If you consent to take part in this study, any of the medical records and data recorded in this study may be directly inspected by representatives of the study Sponsor (TICIPS), the Medicines Control Council of South Africa and other Medicines Regulatory Authorities, the appointed staff at Tijger Trial Centre (e.g. safety monitor, etc), the University of Missouri Health Sciences Institutional Review Board (IRB), Pharma-Ethics Independent Research Ethics Committee, and the Committee for Pharmaceutical Trials of the University of Stellenbosch to make sure that the study is being done correctly. By signing this written informed consent form you are giving permission for this to be done.

The information collected during the study will be stored in a computer but your name will not be stored. Only your study doctor will know that the information is related to you. All blood collected during the study will be labeled with your anonymous subject number and initials. Samples will be sent to a central laboratory for analysis in batches, but your name will not be included on any specimens or accompanying documentation that is sent to the laboratory.

The results of the study may be published in the medical literature and/or presented at a scientific conference or symposium, but your identity will not be revealed. The information disclosed will be collective summarized data.

You may ask to see your medical information as prescribed by law. The treatment that you received in the study needs to remain unknown (blinded) until the study data is analyzed; you may see this information, but only after the data has been analyzed.

**Who is organising and funding the study?**

This research study is being organized and funded by TICIPS who has contracted Tijger Trial Centre to conduct it. Prof J. A. Syce is the project leader and the contact person for TICIPS and Dr Haylene Nell, the clinical investigator responsible for the conduct of the trial and representative of Tijger Trail Centre.

**Who has reviewed the study?**

This study has been approved by the South African Medicines Control Council, the Committee for Pharmaceutical Trials of Stellenbosch University and Independent Research Ethics Committee. The study will also be reviewed by the Institutional Review Board of the University of Missouri, USA (and partner in TICIPS). These are groups of scientific and non-scientific people. They review and approve or disapprove research involving human subjects. The study is in accordance with the guidelines of the International Conference on Harmonization (ICH) for Good Clinical Practice (GCP) and with the Declaration of Helsinki (version 2000). These are policy statements that protect the rights of study patients and volunteers.

If you require details about these committees, you should ask the study doctor (or project leader).

You should inform your general practitioner (your house doctor) that you are taking part in this study. If you have a life insurance policy, you must tell the company involved that you will be taking part in this study.

**Contacts for further information.**

If during the course of this study, you have questions about the nature of the research or your rights, or you believe that you have sustained a research-related injury, you should contact one of the following:

Dr H Nell (study doctor) at +27 21 918 1399/1272 or 083 448 1456

Prof J A Syce at + 27 21 959 2192 or 082 202 3315

If you have any questions about your rights as a participant in a research experiment/trial, you can contact the Committee for Pharmaceutical Trials of the University of Stellenbosch at (021) 938 9075.

If neither the study doctor nor the independent Ethics Committee can assist you, you may write to the Medicines Control Council of South Africa at:

The Registrar

SA Medicines Control Council

# Department van Health

Private Bag X828

PRETORIA

0001

Fax: (012) 323-4474

INFORMED CONSENT FORM

**Patient identification number: . . . . / . . . . . Patient initials:**

**Project title:** A randomized double blind placebo controlled study to investigate the

safety of *Sutherlandia frutescens (subspecies microphylla)* in healthy

adult volunteers.

Protocol number: TICIPS001

By signing and dating this document,

- I confirm that I have had time to carefully read and understand the patient information sheet provided for this study.
- I confirm that I have had the opportunity to discuss the study and ask questions and I am satisfied with the answers and explanations that I have been provided.
- I give permission for my medical records to be reviewed by the Sponsor or designee, and/or representatives of the Medicines Control Council or Committee for Pharmaceutical Trials.
- I understand that my participation is voluntary and that I am free to withdraw at any time without giving any reason and without my medical care or legal rights being affected.
- I confirm that I have received a signed and dated copy of the Patient Information Sheet and Informed Consent Forms.

VOLUNTEER: _________________ _______________ ______________

Name (capital letters) Signature Date

*WITNESS: _________________ _______________ _________________*

*(where required)*

Name (capital letters) Signature Date

*PERSON OBTAINING CONSENT*:

_________________ _______________ ________________

Name (capital letters) Signature Date

*CLINICAL INVESTIGATOR*:

_________________ _______________ ________________

Name (capital letters) Signature Date

**APPENDIX 10.2b**

**PASIëNTE INLIGTINGSTUK EN TOESTEMMINGSVORM**

# PASIëNT INLIGTINKGSTUK EN

# TOESTEMMINGSVORM

**Pasiënt identifikasie nommer: . . . . / . . . . . Pasiënt voorletters:**

**Titel van die studie: ‘n Gerandomiseerde, dubbel blinde, plasebo gekontrolleerde studie om die veiligheid van *Sutherlandia frutescens (subspecies microphylla)* in gesonde volwasse vrywilligers te ondersoek**.

**Protokol nommer:** TICIPS001

**Borg:** The International Centre for Indigenous Phytotherapy Studies (TICIPS)

**Naam van hoof ondersoeker:** Dr H Nell

**Naam van projekleier:** Prof J A Syce

**Adres van plek/instansie waar studie sal plaasvind:** Tijger Trial Centre (TTC), Eerste vloer 1A, Karl Bremer Hospitaal, Bellville, Kaapstad, Suid Afrika & UWK, Studente Gesondheidsorg Sentrum, Bellville, Kaapstad, Suid Afrika.

**INLEIDING**

U word uitgenooi om deel te neem aan ‘n kliniese navorsingstudie wat geborg word deur die **International Centre for Indigenous Phytotherapy Studies (TICIPS).** TICIPS bestaan uit personeel van die Universiteit van die Wes-Kaap, die Universiteit van Missourie, Columbia en die Missourie Botaniese Tuine in die Verenigde State van Amerika wat betrokke is by gesamentlike navorsing op inheemse Afrika plantmedisyne en voedingstowwe. Voordat u besluit om aan die studie deel te neem, is dit belangrik vir u om te verstaan waarom die navorsing gedoen word en wat met u sal gebeur. Hierdie inligtingstuk sal aan u inligting verskaf oor die studie asook u regte as ‘n navorsingsvrywilliger sodat u kan besluit of u wel wil deelneem. Neem asseblief u tyd om noukeurig deur hierdie inligtingstuk te lees en vra die navorser indien iets nie duidelik is nie, of as u verdere inligting verlang.

Wat is die doel van hierdie studie?

Die doel van hierdie studie is om die veiligheid van die plant medisyne ***Sutherlandia frutescens*** te bepaal. Laasgenoemde is die studie medisyne, ‘n term wat later in hierdie dokument en studie gebruik word.

***Sutherlandia frutescens*** is ook bekend as Kankerbos in Afrikaans, Cancer Bush in Engels en Unwele in Zoeloe, ens. Indien dit egter korrek en optimaal gebruik gaan word, sal die veiligheid daarvan akkuraat bepaal moet word en eerstens dan in gesonde vrywilligers. Dit is derhalwe die doel van hierdie studie.

In hierdie studie word *Sutherlandia frutescens (subsp microphyllin*) in kapsule vorm of ‘n soortgelyke kapsule sonder die plant (d.w.s ‘n plasebo) aan 24 gesonde vrywilligers gegee. Hulle sal dan die medisyne twee maal daagliks per mond inneem vir 3 maande terwyl hulle gemonitor sal word vir enige newe effekte van die plantmedisyne.

Die studie sal plaasvind by die Tijger Trial Centre wat by die Karl Bremer Hospitaal geleë is.

**Moet ek aan die studie deelneem?**

Deelname aan hierdie studie is heeltemal vrywillig. Dit is u besluit of u gaan deelneem al dan nie.

Indien u nie belang stel om aan die studie deel te neem nie of aan die studie will onttrek gedurende die studietydperk, hoef u geen rede te verskaf vir u besluit nie en u sal geen voordele verloor waarop u andersins geregtig sou wees nie.

Indien u van die studie sou onttrek, sal die data wat tot by u onttrekking gekollekteer is, geanaliseer word vir die doel van die studie.

Indien u egter besluit om deel te neem aan die studie, sal u hierdie inligtingstuk ontvang en sal u gevra word vir u handtekening, wat u toestemming sal aandui.

**Wie is geskik om aan die studie deel te neem?**

Ongeveer 24 mense (ouer as 18 jaar en jonger as 45) sal aan die studie deelneem. Om te kwalifiseer vir hierdie studie moet u in die verwagte gewiggrense val, nie enige kroniese medikasie gebruik nie, effektief met die studie personeel kan kommunikeer en oor ‘n algemene goeie gesondheid beskik. Afgesien van die feit dat u ‘n gesonde volwassene moet wees, is daar ook ander kriteria om aan die studie deel te kan neem en die studiedokter sal dit nagaan deur aan u sekere vrae te rig asook ‘n fisiese ondersoek te doen.

U mag egter nie aan hierdie studie deelneem indien u nie kan ophou rook vir die tydperk van die studie nie. Die volgende persone mag ook nie aan die studie deelneem nie: as u borsvoed of swanger is; verslaaf is aan dwelmmiddels of ‘n geskiedenis het van dwelmverslawing. Sou u binne ‘n maand voor die begin van hierdie studie deelgeneem het aan enige ander studie, mag u ook nie deelneem nie.

Daar is nog nie voldoende inligting oor die gebruik van *Sutherlandia frutescens* (studie medikasie) in swanger vroue nie en die risiko verbonde aan die menslike ongebore baba is nog onbekend. Aktiewe bestanddele in *Sutherlandia frutescens* mag oor die plasenta beweeg en nadelig wees vir, of wanvorming van, die ongebore baba veroorsaak. Aktiewe bestanddele van *Sutherlandia frutescens* mag ook deur borsmelk geabsorbeer word. Derhalwe word swanger vroue, vroue wat beplan om swanger te word, of wat besig is om kinders te borsvoed, versoek om nie aan die studie deel te neem nie.

**Wat sal met my gebeur indien ek deelneem?**

Indien u besluit om deel te neem, moet u eerstens die toestemmingsvorm teken en dan sal u ondersoek word om te bepaal of u aan al die vereistes van die studie voldoen. Hierdie ondersoeke (Besoek 1) sal die volgende insluit: ‘n mediese geskiedenis wat ook die geskiedenis van die gebruik van medisyne insluit; ‘n fisiese ondersoek; die trek van 40 ml (ongeveer 8 teelepels) bloed vir laboratorium toetse; ‘n EKG (d.i. ‘n Elektrokardiogram wat die aktiwiteite van die hart registreer) en indien u vroulik is, ‘n urine monster vir ‘n swangerskap toets.

Indien u kwalifiseer om by die studie ingesluit te word, sal u binne 14 dae van die eerste ondersoek - by Besoek 2 - u studie medikasie ontvang wat u as volg gaan gebruik: 1 kapsule elk in die oggend en aand na maaltyd. Daar sal egter 2 tipes studie medikasie wees, *Sutherlandia frutescens* en plasebo kapsules, wat identies in voorkoms is en u mag enige een van die twee ontvang. Die toekenning van die kapsule is deur lukraak seleksie en nie u of die studie dokter sal weet watter kapsule u ontvang het nie alvorens die studie voltooi is nie. U het dus ‘n een uit twee kans om die aktiewe studie medikasie te ontvang.

By hierdie besoek (Besoek 2) sal u ‘n maand se voorraad medikasie ontvang, asook ‘n dagboek wat u daagliks moet invul. Die dagboek en ongebruikte medikasie sal u maandeliks tydens u besoek terugbring. In die dagboek sal u die daaglikse gebruik van die studiemedikasie aanteken asook enige newe effekte wat u mag ondervind.

U sal die studiemedikasie vir ‘n volle 3 maande moet gebruik en elke 4 weke na die sentrum terugkeer vir ‘n verdere 3 besoeke. Dit beteken dat die studie tussen 3 en 4 maande sal duur.

Gedurende Besoeke 3 en 5 sal u weer deur die studie dokter ondersoek word en gevra word of u enige mediese probleme ondervind het sedert die vorige besoek. Dieselfde ondersoeke wat gedurende Besoek 1 gedoen was (d.w.s ‘n EKG, trek van 40 ml bloed, ens) sal ook tydens hierdie besoeke herhaal word. U sal egter geen medikasie tydens die laaste besoek (Besoek 5) ontvang nie, aangesien die studie dan voltooi is.

**Wat sal ek moet doen?**

As deelnemer aan hierdie studie, sal u verantwoordelik wees vir die volgende:

- Die nakoming van al die bespreekte kliniekbesoeke
- Gebruik van die studie medikasie soos deur die studiedokter aan u verduidelik;
- Invul van die dagboeke wat u sal ontvang;
- Terugbring van die ingevulde dagboeke tydens elke besoek;
- Aanmelding aan u studie dokter voor u enige ander medikasie begin gebruik;
- Enige verandering van u gesondheid dadelik aan u studie dokter rapporteer;
- **Verseker dat, indien vroulik en u in u vrugbare tydperk is, u alle nodige voorsorgmaatreël sal neem om nie tydens die studie swanger te word nie; en**
- Terugbring van alle ongebruikte medikasie asook die verpakking van alle gebruikte medikasie tydens elke besoek;

Indien u enige beserings sou opdoen en ‘n dokter moet besoek, sal u ook daardie dokter moet meedeel dat u aan hierdie navorsorsingstudie deelneem.

**Watter risikos of ongemak is aan die studie verbonde?**

Soos met alle medisyne, mag u sommige newe effekte ondervind terwyl u die studiemedisyne neem. Trouens die doel van hierdie studie is juis om te bepaal of *Sutherlandia frutesecens* wel enige ongewenste effekte veroorsaak.

Die enigste newe effekte wat tot dusver aangeteken is, wat *Sutherlandia frutesecens* mag veroorsaak, is geringe los stoelgang, geringe hardlywigheid, droë mond en, in rare gevalle, lighoofdigheid by verswakte pasiënte.

Verskeie chemiese verbindings is al in die plant *Sutherlandia frutesecens* geissoleer wat dalk newe effekte mag veroorsaak. Die meeste van die bestanddele mag egter voordelig wees en derhalwe die rede(s) wees waarom hierdie plant effektief in sommige siektetoestande gebruik kan word. Sommige van hierdie potensiële effekte (verlaging van bloed suiker, verhoging van autoimmune siektes) mag by sommige individue ongewens wees. Ons moet ontvanklik wees vir die moontlikheid dat dit wel kan plaasvind. In hierdie studie word die vlakke van sommige van die aktiewe bestanddele spesifiek gemonitor om te bepaal of dit dalk betrokke is by enige newe effekte van die plant medisyne. Oor die algemeen word daar nie verwag dat die plant medisyne ‘n hoë potensiaal het om nadelige reaksies te veroorsaak nie.

Sommige van die prosedures wat tydens die studie uitgevoer word mag ongemak veroorsaak (d.i. die trek van bloed mag geringe kneusing of moontlike floute veroorsaak, u mag ongemak ondervind tydens die neem van die EKG, ens), maar dit sal nie enigsins meer wees as wat u tydens ‘n tipiese mediese ondersoek ondervind nie.

Indien u ‘n vrou in u vrugbare leeftydperk is, mag u slegs aan die studie deelneem indien u ‘n medies aanvaarbare metode van geboorte beperking (voorbehoedmiddel) gebruik.

***Medies aanvaarbare metodes van geboorte beperking sluit die volgende in:*** *orale, binnespierse of subkutane voorbehoedmiddels, intra-uterine toestelle, chirurgiese metodes soos histerektomie of sterilisasie of metodes (soos versperrings metodes, vasektomie by vaste manlike maat, ens) wat volgens die diskresie van die kliniese ondersoeker aanvaarbaar is.* Die studie dokter sal hierdie aangeleentheid by die eerste besoek met u bespreek. Daar sal ook boonop ‘n swangerskap toets by die eerste besoek gedoen word asook elke keer wanneer bloed monsters geneem word. Indien die swangerskap toets positief is, sal die studiedokter dadelik u studiemedikasie staak.

**Wat is die moontlike voordele om aan die studie deel te neem?**

U sal waarskynlik geen direkte voordeel uit u deelname aan die studie verkry nie, maar al die beplande besoeke en ondersoeke om u gesondheid te monitor word gratis aangebied. Die inligting wat ons sal versamel uit die studie sal ons bestaande kennis egter uitbrei en sal ons help om te besluit of die medikasie veilig is om te gebruik deur mense soos u.

**Is daar enige koste verbonde om te kan deelneem?**

Daar sal nie enige fooie deur u betaalbaar wees om te kan deelneem nie, maar u sal genoeg tyd moet toelaat om besoeke aan die kliniek te bring. U sal nie betaal word om deel te wees van die studie nie, maar al die ondersoeke, toetse en studiemedikasie sal gratis aan u verskaf word. Die studiedokter sal u egter vra of u enige vervoerkoste aangegaan het om na die kliniek te kom. U moet al die kwitansies vir bus, trein, huurmotor fooie, ens hou en aan die studiedokter gee. U sal vergoed word vir enige redelike vervoeronkoste wat u mag aangaan as gevolg van u deelname aan die studie.

**Wat as iets verkeerd gaan of indien ek probleme ondervind tydens die studie?**

Indien u ‘n studie verwante besering opdoen, sal u mediese onkostes gedek word asook die behandeling van liggaamlike beserings wat deur die gebruik van die studie medikasie veroorsaak was. ‘n Versekeringspolis wat enige skade aan u gesondheid as gevolg van u deelname aan hierdie studie sal dek is uitgeneem. Vergoeding vir ‘n studie verwante besering sal plaasvind volgens gevestigde standaarde wat deur die *Association for British Pharmaceutical Industry* (ABPI) daargestel is. Die studie dokter sal u ‘n kopie van die ABPI-riglyne gee indien u dit sou verlang.

In geval van ‘n studie verwante besering kontak asseblief:

Dokter: Dr Haylene Nell

Kontak Nommers: +27 21 918 1399/1272 of 083 448 1456

Indien u beseer word as gevolg van iemand se nalatigheid, mag u gronde hê vir regsaksie. U doen nie afstand van u wetlike regte deur hierdie vorm te onderteken nie.

Vir enige verdere inligting rakende die versekeringsdekking, kan u navraag doen by die studiedokter.

**Kan ek onttrek of geskraap word van die studie?**

Deelname aan hierdie studie is vrywillig. Indien u besluit om deel te neem aan die studie, is u geregtig daarop om te eniger tyd van die studie te onttrek. Indien u besluit om u te onttrek, moet u dadelik die studiedokter in kennis stel. U dokter sal nie ontsteld wees of u in enige opsig penaliseer nie en toekomstige sorg sal nie geaffekteer word nie. Wannneer u van die studie onttrek, sal alle data wat voor u onttrekking gekollekteer is, nog steeds as deel van die studie saam met die ander data geanaliseer word.

Omstandighede wat mag opduik en kan lei tot onttrekking van u deelname sluit die volgende in:

- Mediese redes insluitend swangerskap;
- Indien u nie die studie medikasie neem nie of u nie u dagboek voltooi nie of indien u nie u gereëlde afsprake nakom nie of indien u nie die dokter se instruksies volg nie;
- Indien u medikasie neem wat nie in hierdie studie toegelaat word nie;
- Indien daar nie genoegsame pasiënte in die studie is nie;
- Indien die Medisyne Beheer Raad of die Komitee vir Farmaseutiese Studies die navorsing opskort of uitstel of
- Indien die Borg die studie staak.

**Sal my deelname aan die studie vertroulik gehou word?**

Die verslae wat u identifiseer sal vertoulik gehou word en sover die verskeie wette en regulasies dit toelaat, nie openbaar gemaak word nie. Indien u toestemming gee om deel te neem aan die studie, mag enige van die mediese verslae en data ingewin tydens die studie direk geinspekteer word deur verteenwoordigers van die studie Borg (TICIPS), die Medisyne Beheer Raad en ander Medisyne Regulerende Owerhede, die aangeduide personeel by die Tijger Trial Centre (d.i. veiligheids monitor, ens), die University of Missouri Health Sciences Institutional Review Board (IRB), Pharma Ethics Independent Research Ethics komitee en die Komitee vir Middelproewe van die Universiteit van Stellenbosch om te verseker dat die studie korrek gedoen word. Deur hierdie ingeligte toestemming te teken, gee u toestemming vir bogenoemde prosedure.

Die inligting wat gedurende die studie gekollekteer word, sal op ‘n rekenaar gestoor word, maar u naam word nie gestoor nie. Slegs u studie dokter sal weet dat die informasie op u betrekking het. Alle bloedmonsters wat tydens die studie gekollekteer word, word slegs met u anonieme nommer en en voorletters gemerk word. Bloedmonsters word na ‘n sentrale laboratorium gestuur vir analise in groepe, maar u naam sal nie ingelsluit word op enige monsters of dokumentasie wat dit na die laboratorium vergesel nie.

Die resultate van die studie mag in mediese tydskrifte gepubliseer word en/of by wetenskaplike kongresse of simposiums voorgedra word, maar u identiteit sal nie bekend gemaak word nie. Die inliligting wat so beken gemaak word sal kollektiewe opgesomde data wees.

U mag versoek om u mediese inligting te sien, soos voorgeskryf deur die wet. Die behandeling wat u ontvang het tydens die studie moet onbekend (geblind) bly totdat al die studiedata geanaliseer is; u mag hierdie informasie slegs kry nadat die data geanaliseer is.

**Wie reël en befonds hierdie studie?**

Hierdie navorsingstudie word georganiseer en befonds deur TICIPS wat die Tijger Trial Centre gekontrakteer het om die studie uit te voer. Prof J.A. Syce is die projekleier en die kontak persoon vir TICIPS. Dr Haylene Nell, die kliniese navorser, is verantwoordelike vir die uitvoer van die studie en ook die verteenwoordiger vir Tijger Trial Centre.

**Wie het die studie hersien?**

Hierdie studie is goedgekeur deur die Suid Afrikaanse Medisyne Beheer Raad (MBR), die Komitee vir Middelproewe van die Universiteit van Stellenbosch en Pharma-Ethics Independent Research Ethics Committee**.** Die studie word ook hersien deur die Independent Review Board van die Universiteit van Missouri, VSA (‘n vennoot van TICIPS). Hierdie groepe bestaan uit wetenskaplike asook nie-wetenskaplike mense. Hulle ondersoek en keur studies waarby menslike deelnemers betrokke is, goed of af. Hierdie studie word in ooreenstemming met die riglyne van die Internasionale Konferensie van Harmonisering (ICH) for Good Clinical Practice (GCP) en die Deklarasie van Helsinki (2000) uitgevoer. Hierdie is gedragsriglyne wat die regte van studie pasiënte en vrywilligers beskerm.

Indien u besonderhede van hierdie komitees verlang, kan u die studiedokter of projekleier daarom vra.

Dit is wenslik dat u u algemene praktisyn (huisdokter) meedeel dat u aan hierdie studie deelneem. Indien u ‘n lewensversekerings polis het, moet u die maatskappy verwittig dat u aan hierdie studie gaan deelneem.

**Kontak nommers vir verdere inligting.**

Indien u gedurende die studie tydperk vrae van enige aard rakende die aard van die navorsing of u regte het, of indien u van mening is dat u ‘n navorsings verwante besering opgedoen het, moet u een van die volgende persone kontak:

Dr H Nell (studie dokrter) by +27 21 918 1399/1272 of 083 448 1456

Prof J A Syce by + 27 21 959 2192 of 082 202 3315

Vir enige vrae oor u regte as deelnemer aan ‘n navorsings eksperiment/studie kontak

Die Komitee vir Middelproewe van die Universiteit van Stellenbos by (021) 938 9075.

Indien nie die studie dokter of die onafhanklike Etiese Komitee u van hulp kan wees nie, kan u skryf aan die Medisyne Beheer Raad van Suid Afrika by:

Die Registrateur

SA Medisyne Beheer Raad

# Department van Gesondheid

Privaat Sak X828

PRETORIA

0001

Faks: (012) 323-4474

**INGELIGTE TOESTEMMINGS VORM**

**Pasiënt identifiksie nommer: . . . . / . . . . . Pasiënt voorletters:**

**Projek titel:** ‘**n Gerandomiseerde, dubbel blinde, plasebo gekontrolleerde studie om die veiligheid van *Sutherlandia frutescens (subspecies microphylla)* in gesonde volwasse vrywilligers te ondersoek**.

Protokol nommer: TICIPS001

Deur hierdie dokument te onderteken en dateer,

- Bevestig ek dat ek genoegsame tyd gehad het om die pasiënt inligtinstuk van hierdie studie noukeurig deur te lees en te verstaan.
- Bevestig ek dat ek die geleëntheid gehad het om die studie te bespreek en vrae te vra en dat ek tevrede is met die antwoorde en verduidelikings wat aan my verskaf is.
- Gee ek toestemming dat my mediese verslae nagegaan mag word deur die Borg of ‘n afgevaardigde en/of verteenwoordigers van die Medisyne Beheer Raad of die Komitee vir Farmaseutiese Studies.
- Verstaan ek dat my deelname vrywillig is en dat ek geregtig is om te onttrek van die studie ter enige tyd sonder dat ek redes moet verskaf of dat my mediese of wettige regte geaffekteer sal word.
- Bevestig ek dat ek ‘n getekende en gedateerde kopie van die Pasiënt Inligtingstuk en Toestemmingsvorm ontvang het.

VRYWILLIGER: _________________ _______________ ______________

Naam (hoofletters) Handtekening Datum

*GETUIE: _________________ _______________ _________________*

*(indien nodig)*

Naam (hoofletters) Handtekening Datum

*PERSOON WAT TOESTEMMING VERKRY*:

_________________ _______________ ________________

Naam (hoofletters) Handtekening Datum

KLINIESE NAVORSER:

_________________ _______________ ________________

Naam (hoofletters) Handtekening Datum

###### APPENDIX 10.3a

DIARY (English version) FOR TREATMENT PERIOD

# PATIENT DIARY

**Patient Study Number: ……….. Diary number: …….**

| **Day & date** | **Time of morning dose** | **Taken with meal?** | **Time of evening dose?** | **Taken with meal?** | **List adverse effects / changes in health and level of intensity** | **List any other medication taken** |
| --- | --- | --- | --- | --- | --- | --- |
| *e.g.* 01 Mar 04 | *e.g.*  07:30 | *e.g.*  Yes or No | *e.g.* 19:00 or 7 pm | *e.g.*  Yes or No | *e.g.* As explained by study doctor and see *** below | *e.g.* 2 “Name” tablets; time and reason. |
| 1 |  |  |  |  |  |  |
| 2 |  |  |  |  |  |  |
| 3 |  |  |  |  |  |  |
| 4 |  |  |  |  |  |  |
| 5 |  |  |  |  |  |  |
| 6 |  |  |  |  |  |  |
| 7 |  |  |  |  |  |  |
| 8 |  |  |  |  |  |  |
| 9 |  |  |  |  |  |  |
| 10 |  |  |  |  |  |  |
| 11 |  |  |  |  |  |  |
| 12 |  |  |  |  |  |  |
| 13 |  |  |  |  |  |  |
| 14 |  |  |  |  |  |  |
| 15 |  |  |  |  |  |  |
| 16 |  |  |  |  |  |  |
| 17 |  |  |  |  |  |  |
| 18 |  |  |  |  |  |  |
| 19 |  |  |  |  |  |  |
| 20 |  |  |  |  |  |  |
| 21 |  |  |  |  |  |  |
| 22 |  |  |  |  |  |  |
| 23 |  |  |  |  |  |  |
| 24 |  |  |  |  |  |  |
| 25 |  |  |  |  |  |  |
| 26 |  |  |  |  |  |  |
| 27 |  |  |  |  |  |  |
| 28 |  |  |  |  |  |  |
| 29 |  |  |  |  |  |  |
| 30 |  |  |  |  |  |  |

**** Please assess the intensity of the problem or adverse event as follows:

**Mild = aware of sign or symptom, but it is easily tolerated**

**Moderate = discomfort sufficient to cause interference with normal activities**

**Severe = it is incapacitating, with inability to perform normal activities.**

###### APPENDIX 10.3b

DIARY (Afrikaans version) FOR TREATMENT PERIOD

# PASIëNT DAGBOEK

**Pasiënt Studie Nommer: ……….. Dagboek nommer: …….**

| Dag &  **Datum** | **Tyd van oggend dosering** | **Geneem met ete?** | **Tyd van aand**  **dosering** | **Geneem met ete?** | **Lys ongewenste effekte/ verandering in gesondheid en intensiteitsgraad** | **Lys ander medikasie geneem** |
| --- | --- | --- | --- | --- | --- | --- |
| *bv.* 01 Mar 04 | *bv.*  07:30 | *bv.*  Ja of Nee | *bv*. 19:00 of 7 nm | *bv.*  Ja of Nee | *bv*. Vra studiedokter & vir graad sien *** | *bv.* 2 Panado tablette; tyd en rede |
| 1 |  |  |  |  |  |  |
| 2 |  |  |  |  |  |  |
| 3 |  |  |  |  |  |  |
| 4 |  |  |  |  |  |  |
| 5 |  |  |  |  |  |  |
| 6 |  |  |  |  |  |  |
| 7 |  |  |  |  |  |  |
| 8 |  |  |  |  |  |  |
| 9 |  |  |  |  |  |  |
| 10 |  |  |  |  |  |  |
| 11 |  |  |  |  |  |  |
| 12 |  |  |  |  |  |  |
| 13 |  |  |  |  |  |  |
| 14 |  |  |  |  |  |  |
| 15 |  |  |  |  |  |  |
| 16 |  |  |  |  |  |  |
| 17 |  |  |  |  |  |  |
| 18 |  |  |  |  |  |  |
| 19 |  |  |  |  |  |  |
| 20 |  |  |  |  |  |  |
| 21 |  |  |  |  |  |  |
| 22 |  |  |  |  |  |  |
| 23 |  |  |  |  |  |  |
| 24 |  |  |  |  |  |  |
| 25 |  |  |  |  |  |  |
| 26 |  |  |  |  |  |  |
| 27 |  |  |  |  |  |  |
| 28 |  |  |  |  |  |  |
| 29 |  |  |  |  |  |  |
| 30 |  |  |  |  |  |  |

***** Bepaal asseblief die intensiteit van die probleem of ongewenste reaksie as volg:**

**Matig = u was bewus van teken of simptoom, maar dit was maklik verdra**

**Middelmatig = ongemak was genoeg om normale aktiwiteite te beïnvloed**

**Ernstig = het u onbevoeg gestel om normale aktiwiteite te kan doen**

APPENDIX 10.4

IDEAL BODY MASS TABLES

# Published Literature on Sutherlandia

# Literature attesting to Historical Use

1. Anonymous. 1993. Krueierate van die Montagu Museum. Publication of the museum and board of trustees. (Translation of title: “Herbal Remedies of the Montagu Museum”)
2. van Wyk, B.-E., van Oudtshoorn, B. and Gericke, N. 1997. Medicinal Plants of South Africa. Briza, Pretoria.
3. van Wyk, B.-E. and Gericke, N. 2000. People’s Plants. A Guide to the Useful Plants of Southern Africa. Briza, Pretoria.
4. Rood, B. 1994. Uit die Veldapteek. Tafelberg, Cape Town. (Translation of title: “Out of the field-pharmacy”).
5. Von Koenen, E. 1996. Heil- Gift und Essbare Pflanzen in Namibia. Edition Namibia. Klaus Hess Verlag. (Translation of title: Medicinal, Poisonous and edible plants of Nambia).
6. Watt, J.M. and Breyer-Brandwijk, M.G. 1962 (2nd edition). The Medicinal and Poisonous Plants of Southern and Eastern Africa. E&S Livingstone Ltd., Edinburgh.
7. Pappe, L. 1857. Florae Capensis Medicae Prodromus. 2nd Edition. Brittain, Cape Town.
8. Smith, C.A. 1966. Common Names of South African Plants. Botanical Survey Memoir No. 35. Government Printer, Pretoria.
9. Roberts, M. 1990. Indigenous Healing Plants. Southern Book Publishers, Halfway House.

# Literature: Chemistry and Pharmacology

1. Crooks, P.A. and Rosenthal, G.A. (Filed Dec 5, 1994) Use of L-canavanine as a chemotherapeutic agent for the treatment of pancreatic cancer. United States Patent 5,552,440.
2. Green. M.H. (Filed Jan 25, 1988) Method of treating viral infections with amino acid analogs. United States Patent 5,110,600.
3. Narayanan, et al. (1987) Pinitol - A New Anti-Diabetic compound From the Leaves of *Bougainvillea Spectabilis*. Current Science 56(3), 139-141.
4. Ostlund, R.E and Sherman, W.R (Filed March 4, 1996). Pinitol and derivatives thereof for the treatment of metabolic disorders. US Patent 5,8827,896.
5. Swaffar, D.S. et al. (August 1995) Combination therapy with 5-fluorouracil and L-canavanine: in-vitro and in-vivo studies. Anticancer Drugs, 6(4), 586-93.
